# Analysis of public records of lobbying practices of the ultra-processed sugary food and drink industries in Chile: a qualitative study

**DOI:** 10.1016/j.lana.2024.100794

**Published:** 2024-06-09

**Authors:** Yanela Aravena-Rivas, Anja Heilmann, Richard G. Watt, Tom Broomhead, Georgios Tsakos

**Affiliations:** a Department of Epidemiology and Public Health, University College London, 1-19 Torrington Place, London, WC1E 7HB, UK; b Unit of Oral Health, Dentistry and Society, School of Clinical Dentistry, University of Sheffield, 19 Claremont Crescent, Sheffield, S10 2TA, UK

**Keywords:** Chile, Sugar, Health policy, Lobbying, Commercial determinants of health

## Abstract

**Background:**

Given the role of commercial determinants on sugar consumption and health, this study aimed to describe lobbying practices of the ultra-processed sugary food and drinks industries in Chile between 2014 and 2022.

**Methods:**

Official meetings between ultra-processed sugary food and drinks industries and related commercial actors and Chilean government officials were obtained from the Chilean Lobby Registry. Relevant commercial names were initially identified based on their market share and expanded iteratively based on information from relevant meetings. Qualitative analysis followed a deductive-inductive approach using the Corporate Political Activity Model to identify and classify objectives, framing and action strategies.

**Findings:**

From 237 records identified, the Ministries of Health, Social Development, and Economy were the most frequently lobbied. Industry representatives sought to achieve their short- and long-term objectives by targeting a diverse range of authorities, including Ministers and Under-secretaries, using different strategies. Framing strategies focused on presenting sugary food and drinks industries as socially responsible and legitimate policy actors and criticised public health initiatives as ‘bad solutions’. Action strategies aimed to influence policymaking and nurture corporate reputations.

**Interpretation:**

Extensive lobbying took place by the sugary food and drinks industries between 2014 and 2022, a period when major public health policies were being discussed in Chile. Lobbying strategies varied to meet industry objectives and targeted a diverse range of government institutions including high-ranking officials. Tighter regulations to stop inappropriate industry influence in public health policymaking are urgently required.

**Funding:**

10.13039/501100020884Agencia Nacional de Investigación y Desarrollo (Chile)-PhD Scholarship. 10.13039/501100000765University College London–Open Access fees.

**Research in context**

**Evidence before this study**

A PubMed search on Apr 26, 2024, using the following MeSH terms: “Food Industry” AND (“Chile” OR “Latin America”) AND “Health Policy”, identified 47 papers of which only two explored corporate political practices (including lobbying) of the food and drinks industry in Chile. Both studies highlighted the increasing influence of food and drinks industries on policymaking and the use of diverse corporate political practices and called for further monitoring of these practices and the development of more robust mechanisms to address their influence. However, these studies were either broad in their scope (focusing on corporate political practices in general), used a narrower timeframe, or classified activities based on older frameworks that have recently been updated, and therefore did not expand on the lobbying strategies used by ultra-processed sugary food and drinks industries.

**Added value of this study**

This study described lobbying practices of ultra-processed sugary food and drink industries and related commercial actors in Chile since the implementation of the National Lobby Registry in 2014 and during a period of political discussions and implementation of major structural public health policies in nutrition (e.g., front-of-package labelling, advertising restrictions, sugar-sweetened beverage tax), expanding on what previous studies had described. The study also used a novel framework to classify corporate political activities providing a new perspective on lobbying practices in Chile by including new categorisations for framing and action strategies as well as considering long and short-term objectives. The findings documented the frequent, bold, and inappropriate use of lobbying by these industries to influence public policies according to their interests.

**Implications of all the available evidence**

The frequency of lobbying practices in Chile implies the need for effective regulation mechanisms against inappropriate lobbying of authorities and officials who develop and implement public health policies. This is particularly relevant in the context of this study's findings that showed that lobbying has often been used to hinder evidence-based whole-population approaches or promote industry-friendly individual-level activities. The diversity of claims and actions used by these commercial actors, and their bold shape in the Chilean context may also be indicative of lobbying practices in the Latin American region, raising the need for bespoke measures based on specific political and cultural contexts.

## Introduction

Unhealthy diets are a major risk factor for several globally prevalent health conditions. High sugar consumption, a central aspect of an unhealthy diet, is associated with obesity,^1^, ^2^, ^3^ type 2 diabetes,^4^^,^^5^ cardiovascular disease,^6^ and dental caries.^7^ These conditions disproportionately affect more vulnerable populations and are significant global public health problems due to their long-lasting cumulative adverse effects, impact on quality of life, and economic burden for individuals and societies.^8^ However, reducing sugar intake at a population level has proven to be enormously difficult.^9^

Structural social determinants such as cultural, political and economic systems shape the accessibility, availability and affordability of foods and drinks,^10^ and ultimately influence dietary behaviours and their impact on health. Commercial actors have been shown to strongly influence these determinants, and this has had a negative impact on population health and equity.^11^ These “systems, practices, and pathways through which commercial actors drive health and equity”^11^ are known as the commercial determinants of health. Ultra-processed food industries spend substantial resources to create systems that maximise profit and resist change through public health interventions by applying a complex web of practices based on their business models and growth strategies.^12^ These practices, known as corporate political activities, aim “to secure preferential treatment and/or prevent, shape, circumvent or undermine public policies in ways that further corporate interests”.^13^

The recently published framework for commercial determinants of health by Gilmore et al.^11^ identifies power as a key underlying driver of the complex interplay between commercial determinants, social determinants, and health. Commercial entities can operationalise their power through practices such as lobbying and shape norms in ways that prioritise their interests over health or social equity, negatively impacting health.^11^ Political science research has shown the long-standing influence of large businesses over the Chilean state in the past decades, including their support for the military regime that established the strong free market economy currently in place, and its influence on the current democratic system.^14^^,^^15^ Their political pragmatism, placing economic benefit above all else, and their access to policymakers both through their relevance as corporate entities and personal ties to authorities, have been identified as key elements for their effectiveness in influencing governments from different political parties.^14^^,^^15^

**Fig. 1:**
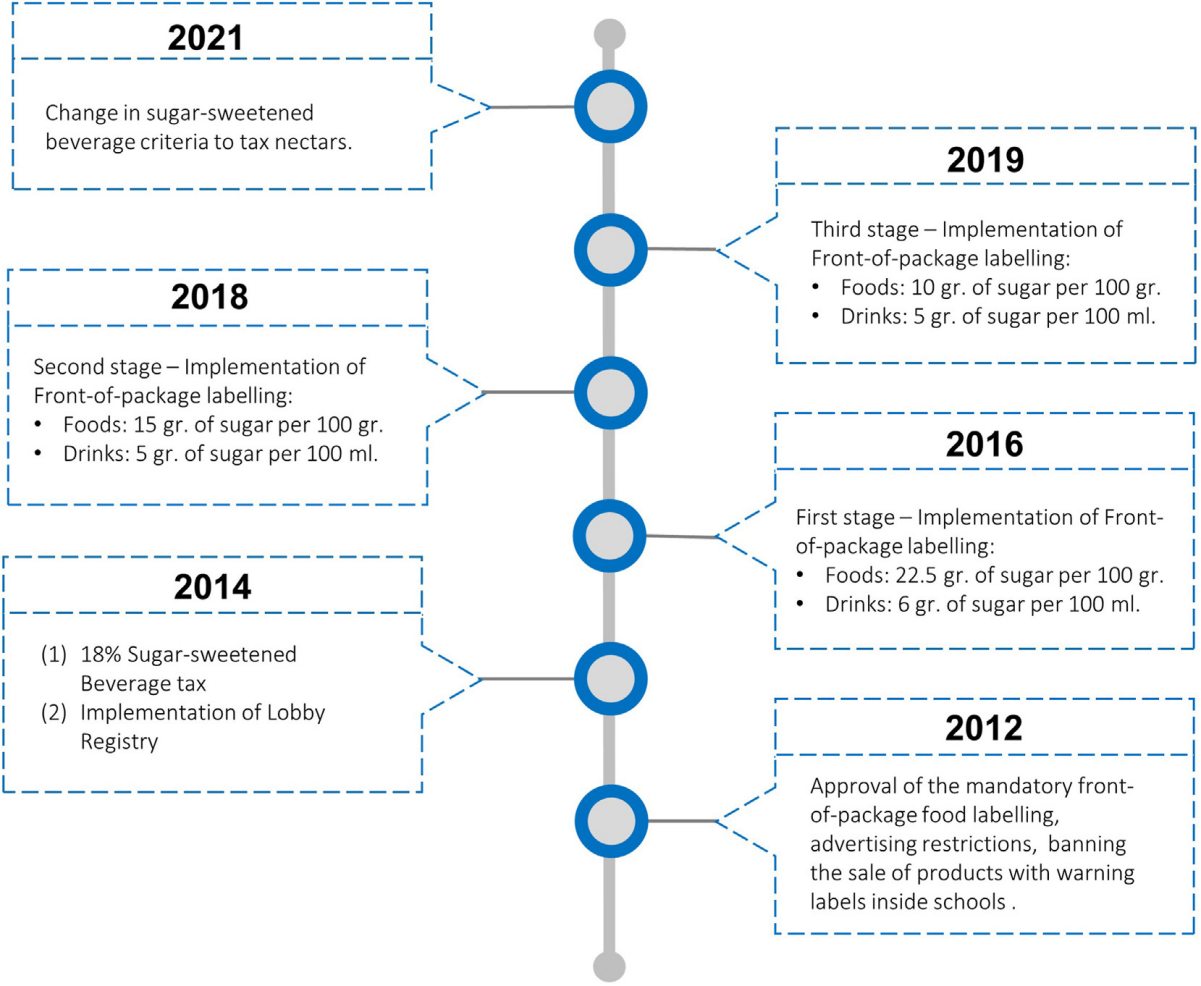
Timeline of key public health policies to create healthier food systems implemented Chile.

In this context, the ultra-processed sugary food and drink industries have strongly influenced food systems in Latin America and the Caribbean. Mialon and collegues^16^ identified more than 200 examples of corporate political activities in the region. Industry strategies have been largely based on lobbying policymakers to influence public health regulations, highlighting the importance of the industry to the local economy, using their platforms to divert attention from their products as causes of disease, and building alliances with health professionals, universities, and local communities. In Chile, a range of corporate political activities have also been identified, including interactions with national researchers via sponsorship of events, social activities for children and schools, and direct lobbying of policymakers.^17^ However, there is still limited understanding of how lobbying practices of ultra-processed sugary food and drink industries have been used to obtain and exert corporate power, influence decision making, and ultimately affect the food system. This is particularly relevant considering the role of commercial actors in influencing dietary behaviours, particularly in the current context where many low- and middle-income countries in the region are discussing public health policies aiming to create healthier food systems such as mandatory front-of-package labelling regulations and sugar-sweetened beverage taxes. Both these policies are recommended as ‘best buys’ by the Pan American Health Organisation,^18^ and have already been implemented in Chile in recent years: front-of-package warning labels, advertising restrictions, and banning the sale inside schools of products with warning labels were approved by Congress in 2012 and implemented in three stages, while a sugar-sweetened beverage tax was implemented in 2014 (a more detailed timeline can be seen in Fig. 1). Thus, a deeper understanding of corporate political activities in Chile can also provide insights for other countries in the region, such as Colombia, Brazil, and Argentina, going through similar processes where similar strategies are used to oppose these types of policies.^19^ Since 2014, Chile has implemented a mandatory National Lobby Registry^20^ that allows this issue to be explored with more concrete empirical evidence. This study aims to describe the lobbying practices of the ultra-processed sugary food industry in Chile using the information provided by the National Lobby Registry between 2014 and 2022.

## Methods

### Data sources and search strategy

Data were obtained from official meeting records. These have been mandatory since 2014, following the law to manage lobbying in Chile (Law n° 20,730) approved by the National Congress.^21^ Meetings with government officials can be requested online or in person by filling out a form. Authorities have three working days to accept or deny the request. Each institution keeps records of its meetings (through a Lobby Registry) and displays them on its websites.

The Lobby Registry is open to the public and was implemented in three stages between 2014 and 2015 to include central, regional, and local (i.e., municipal) government. The registry keeps records of meetings requested by any natural or legal person including representatives from private entities and civil society, both paid (i.e., lobbyists) and non-paid (i.e., ‘private interests’ managers), for the purpose of influencing public decisions. Topics that can be discussed include administrative activities, draft bills, laws, contracts, regulations, programmes, and Congress agreements and decisions.^22^ Travel and gifts received by authorities and public servants are also recorded and added to the registry. All records from different institutions' lobby registries are collected and made available on two websites.^22^ One is the Chilean Platform for Lobbying Registry ‘LeyLobby’^23^ managed by the Ministry General Secretariat of the Presidency, built to register official meetings by different public institutions. The other is ‘InfoLobby’^24^ managed by the Chilean Transparency Council, built to store and facilitate access to records of lobbying activities in Chile.

In this study all meeting requests made by industries that make ultra-processed sugary products (including those with broader product portfolios) and related corporate interest groups were included from 2014 (implementation of the registry) to 2022. To identify relevant corporate interest groups, the classification proposed by Slater et al.^25^ was used. They classify corporate interest groups related to the general food industry into those focused on i. ‘primary production processing and ingredients’; ii. ‘food manufacturing and retail’; iii. ‘branding and advertising’; iv. ‘general business and trade’; v. ‘research and science communication’; vi. ‘lobbying, legal and public relations’; vii. ‘sustainability’; viii. ‘specialised nutrition and baby food’; and ix. ‘others’. Names of identified interest groups can be found in the Supplementary Methods. Data collection was conducted between October 2022 and August 2023. The identification of the relevant meetings was carried out in sequential methodological stages. First, the most important industry actors (based on their market share) and major national food-related trade associations were identified.^26^ Second, a search based on these names was conducted to collect relevant meeting records. Third, other commercial actors were identified using the names and affiliations of those participating in the meetings selected in the second stage. This iterative process continued until no new names or affiliations were found. In the fourth stage, a search using keywords based on the most common topics discussed at these meetings was also conducted (Supplementary Methods). Meetings that were part of the established tender process to bid for products and services to the state were excluded from the analysis, as were meetings discussing specific policies not directly related to nutritional strategies (e.g., recycling regulations).

This study was a qualitative analysis of meeting records in the public domain. Thus, approval by an ethics committee was not required.

### Data analysis

Extracted data were charted in an Excel table. The table included the year, duration of the meeting, number and affiliation of participating lobbyists, position and institution of the public authority or official, and the topics discussed. The qualitative analysis took a realist position and followed a deductive-inductive approach led by the first author (YA). The Corporate Political Activity Model^13^ was used to guide the analysis for a mainly deductive approach as it allowed for the systematic documentation of the characteristics of lobbying activities based on a comprehensive evidence-based framework. The model was developed by following a review of previous models on corporate political practices and with inputs from experts and stakeholders to provide an evidence-based taxonomy applicable to a range of unhealthy commodity industries globally. The model classifies corporate political activities in five framing strategies (i. ‘the ‘good’ actor: corporations'; ii. ‘the ‘bad’ actors: proponents of whole-population, statutory policies'; iii. ‘the ‘trivial’ and ‘individual’ problem: created by a minority of consumers'; iv. ‘the acceptable ‘good’ solution: individual-focused, corporate supported’; v. ‘the unacceptable ‘bad’ solution: whole population, statutory’) and six action strategies (i. ‘access and influence policy-making’; ii. ‘use the law to obstruct policies’; iii. ‘manufacture public support for corporate positions’; iv. ‘shape evidence to manufacture doubt’; v. ‘displace and usurp public health’; vi. ‘manage reputations to corporate advantage’), which are further subdivided into mutually exclusive explanatory categories (19 claims and 21 mechanisms respectively). The model also includes five short- and long-term objectives sought by industry actors when using these strategies. Short-term objectives are defined as those targeting specific policy ‘problems’, while long-term objectives aim to create a friendly policy environment for corporations. Further details of the model can be found elsewhere.^13^

Codes based on the model's objectives, claims and mechanisms were used to identify objectives, framing and action strategies respectively. When the model-based codes were not appropriate for the short description provided by the records, an inductive approach was conducted, and new categories proposed. The codes and categories were discussed between all authors (YA, GT, RW, AH and TB) to define the final categories and their interpretations. One or more objectives, claims or mechanisms per meeting were identified in the present study (e.g., if industries proposed changes to a public health policy and sought to create a public-private alliance for future policymaking, two objectives were identified). In addition, a new claim for ‘the ‘bad’ solution’ framing strategy and a new long-term objective were identified and added to the model, together with new examples of existing categories. There were 65 records without sufficient topic description (e.g., ‘law 20,606’, ‘right to food’, ‘food labelling’, ‘greetings’, etc.) to be classified in a specific category, so these were grouped within the more relevant wider strategy or objective type where possible or excluded from classification.

Summary tables for framing, action strategies, and objectives were created, and the narrative of the results presents illustrative examples following the Corporate Political Activity model classification.

### Reflexivity

The authors are researchers in public health, and this implies an underlying perspective of prioritising public health over economic interests. This influenced the theoretical underpinnings, methods and the chosen framework for this study. The key findings are presented based on a comprehensive evidence-based theoretical model to explore corporate political activity, and the results are discussed in light of the wider literature on the topic.

### Role of the funding source

ANID (Agencia Nacional de Investigación y Desarrollo - Chile) funds the first author's PhD Scholarship (ID 72210219). University College London funds have been used to cover open access publication fees. The funders had no role in study design, data collection, data analysis, data interpretation, writing of the manuscript, or the decision to submit for publication.

## Results

A total of 237 meeting records were identified. The ultra-processed sugary food and drinks industries that lobbied the most were Nestle (43 meetings) and CCU (United Brewery Company–29 meetings). Two national food industry trade associations, AB Chile and Chilealimentos, also lobbied frequently with 32 and 24 meetings respectively (Table 1). Related corporate interest groups lobbying the Chilean government came from other economic sectors such as advertising and general commerce, but also from non-economic sectors such as the Embassy of Italy supporting an Italian company (Ferrero) in meetings discussing the front-of-package labelling regulations. The government bodies lobbied the most were the Ministries of Health, Social Development, and Economy. Local municipal authorities were also frequently lobbied, particularly in locations where these industries have plants or distribution centres. Lobbying targeted a diverse range of authorities and officials (Supplementary Table S1), several of them in high positions of power within government (47 meetings with Ministers or Under-secretaries) or Congress (eight meetings with Congress members). More information on industry actors and representative characteristics, civil servants and topics discussed can be found in the Supplementary Table S1.

**Table 1:** Summary of official meeting records for commercial actors in Chile between 2014 and 2022.

Commercial actor	Government body	Number of official meetings	Total number of official meetings
Nestlé Chile S.A.	Ministry of Health	7	43
	Ministry of Work	4	
	Ministry of Foreign Affairs	3	
	Ministry of Agriculture	3	
	Ministry of Economy	2	
	Ministry of Education	2	
	Ministry of Science	1	
	Ministry of Social Development	1	
	National Consumer Service	1	
	National Training and Employment Service	1	
	National School Aid and Scholarship Board	1	
	National Youth Institute	2	
	Regional Government Authority	3	
	Regional Health Authority	1	
	Local (Municipality) Authorities	11	
Coca-Cola Chile S.A.	Ministry of Health	5	18
	Ministry of Economy	2	
	Treasury	1	
	Ministry of Women and Gender Equity	2	
	Ministry of the Environment	1	
	Ministry of Social Development	1	
	Foreign Investment Promotion Agency	1	
	Regional Government Authority	2	
	Local (Municipality) Authorities	3	
Empresas Carozzi S.A.	Ministry of Social Development	6	21
	Ministry of Health	3	
	Ministry of Foreign Affairs	1	
	Ministry of Sports	1	
	Ministry of Economy	1	
	Treasury	1	
	National Consumer Service	2	
	National School Aid and Scholarship Board	1	
	Local (Municipality) Authorities	5	
CCU S.A. (United Breweries Company)	Ministry of the Environment	3	29
	Ministry of Health	1	
	Ministry of Economy	1	
	Ministry of Culture and the Arts	1	
	National Council for Culture and the Arts	1	
	Regional Government Authority	1	
	Local (Municipality) Authorities	21	
Masterfoods Chile Alimentos Ltda.	Ministry of Health	3	5
	National Consumer Service	1	
	Regional Government Authority	1	
Red Bull	Ministry of Health	4	9
	Ministry of Culture and the Arts	2	
	Ministry of Economy	1	
	National Consumer Service	2	
Arcor Foundation Chile	Ministry of Education	4	7
	Ministry of Sports	3	
Ferrero S.A.	Ministry of Foreign Affairs	2	3
	Ministry of Economy	1	
Grupo Bimbo/Ideal S.A.	Ministry of Foreign Affairs	1	9
	Ministry of Economy	1	
	Local (Municipality) Authorities	7	
La Fete Chocolat/Chocolates del Mundo S.A.	Ministry of Health	2	6
	Ministry of Foreign Affairs	1	
	Ministry of Economy	1	
	Lower Chamber of Congress	2	
PepsiCo	Ministry of Environment	1	4
	Ministry of Education	1	
	Local (Municipality) Authorities	2	
Danone	Ministry of Health	3	3
Soprole	Local (Municipality) Authorities	1	1
Watt's S.A.	Ministry of Education	1	1
Tresmontes Lucchetti S.A.	National Indigenous Development Corporation	1	1
Unilever Chile	Ministry of Health	2	2
Chilealimentos A.G. (Chilean Food Industries Trade Association)	Ministry of Health	8	24
	Ministry of Social Development	7	
	Ministry of Agriculture	2	
	Ministry of Foreign Affairs	2	
	Ministry of Science	1	
	Ministry of Economy	1	
	Treasury	2	
	Solidarity and Social Investment Fund	1	
AB Chile A.G. (Chilean Food and Drinks Industries Trade Association)	Ministry of Health	15	32
	Ministry of Economy	5	
	Ministry of Foreign Affairs	2	
	Ministry of Social Development	1	
	Treasury	2	
	Ministry Secretary of the Presidency	1	
	Tax Office	1	
	National Service for Older Adults	1	
	Constitutional Convention	4	
Santiago Chamber of Commerce	Ministry of Health	2	2
Chilean Federation of Industries (SOFOFA)	Ministry Secretary of the Presidency	1	2
	Ministry of Health	1	
National Advertisers Association (ANDA)	Ministry of Health	4	11
	Ministry of Economy	1	
	Upper Chamber of Congress	4	
	Lower Chamber of Congress	2	
International Life Science Institute (ILSI) Sur Andino	Ministry of health	1	1
Supermarkets Union Association	Ministry of Health	1	1
Italian Embassy	Ministry of Health	1	1

### Framing strategies

Table 2 presents the framing strategies identified. A large number of meetings (83%) focused on presenting ultra-processed food industries as ‘good actors’. This commonly involved sharing their corporate social responsibility strategies (e.g., education in healthy ‘lifestyles’, recycling programmes, and animal welfare) and inviting government authorities to be part of them (e.g., Coca Cola Chile presenting their work with the community to a local mayor, proposing to sponsor a preschool and to help with social activities). In addition, claims highlighting their role in the economy and social fabric were also observed (e.g., Ferrero stressing their 31 years of activity in Chile and their four plants), in several cases during the same meeting (e.g., Nestle introducing a new plant together with projects to support the local community). Industries also presented themselves as legitimate policy and scientific actors, for example, the trade association AB Chile asking the Ministry of Foreign Affairs ‘why it was taking so long to include the private sector in the discussion of public policies’, or Nestle presenting their ‘innovation, science and technology in Chile’ plan to the Minister of Science.

**Table 2:** Framing strategies used by commercial actors during lobbying activities in Chile between 2014 and 2022.

Strategies	Claims	Number of meetings
The ‘good’ actor: corporations	Businesses are legal entities	–
	Industry is key economic actor	21
	Industry is part of the social fabric	8
	Industry is legitimate policy actor	22
	Industry is legitimate scientific actor	12
	Industry is champion of public health	57
	Industry is socially responsible	68
	Industry is victim	–
	Claim not classified	8
The ‘bad’ actors: proponents of whole-population, statutory policies	Policymakers who support unfavourable policies have questionable skills and motives	–
	Public health community have questionable skills and motives	–
The ‘trivial’ and ‘individual’ problem: created by a minority of consumers	Health harms are not caused by Industry's products/services	–
	Health harms arise from consumption patterns of atypical minorities	–
	Health harms are exaggerated	–
The acceptable, ‘good’ solution: individual-focused, corporate supported	Solutions should target individuals, not whole populations	6
	Solutions should be self-regulatory & not disrupt business	–
The unacceptable, ‘bad’ solution: whole population, statutory	Policies are unnecessary & unacceptable	–
	Policies/policy formulation contravene norms, rules & laws	2
	Policies will lead to losses for businesses, economy & society	10
	Policy will fail & have perverse consequences	12
	Policy is ill-thought-out^a^	11
	Claim not classified	16
Strategy not classified		29

a Claim added to the original Corporate Political Activity Model.^13^

In 57 meetings, actors specifically presented themselves as public health champions, for example the Chilealimentos trade association expressing their concerns regarding the increase in non-communicable diseases, their desire to contribute to public policies tackling obesity, and offering their support to public institutions. However, these supportive arguments were often presented together with criticisms of public health initiatives. For example, AB Chile stated that they were ‘available for anything the Ministry of Health needed’, while at the same time arguing that ‘the front-of-package labelling regulations were not effective’ when the available evidence pointed in the opposite direction.

Other meetings (n = 51) had the purpose of discussing specific policies as a ‘bad solution’. These focused on the perceived negative impact on businesses and the economy (e.g., the food company Carozzi stating that the front-of-package labelling regulations were ‘limiting free trade, hindering national exports and being seriously detrimental to private investment’), or their ‘poor implementation’ (e.g., Carozzi proposing improvements to ‘correct perceived inconsistencies of significant health relevance’ of the front-of-package regulations). Examples of lobbying for individual ‘lifestyle’ interventions as part of ‘the acceptable ‘good’ solution’ strategy were also observed, such as the Chilealimentos trade association inviting officials from the Ministry of Social Development to join in a national agreement to ‘strengthen healthy lifestyles’.

The Corporate Political Activity model was expanded by adding a new claim, which grouped together 11 meetings asking questions regarding the implementation of the front-of-package labelling regulations. This category was named ‘policy is ill-thought-out’ as industries indirectly expressed their concerns regarding public policies (e.g., Nestle requesting a meeting at the Ministry of Health to discuss ‘unclear regulation guidelines’). No examples of the categories ‘the trivial and individual problem’ and ‘the ‘bad’ actors' were observed. Twenty-four meetings could only be classified within the broader strategy category due to a lack of details. Furthermore, 29 meetings could not be classified under any framing strategy due to their very brief description in the records.

### Action strategies

Examples for all six action strategies were identified (Table 3). The largest number of meetings (51%) aimed to ‘access and influence policymaking’ in different ways. Industries used international spaces to seek access (e.g., Chilealimentos requesting participation in policymaking spaces based on a previous agreement made during an Organization for Economic Co-operation and Development meeting) or directly requested them from authorities (e.g., Carozzi highlighting ‘the need for joint work between the public and private sectors to move towards common food labelling’). Furthermore, industries attempted to influence policymaking by directly approaching authorities to share their concerns and proposals to modify the legislation (e.g., the National Association of Announcers (ANDA) presented their concerns regarding the implementation of the front-of-package labelling regulations claiming some elements did not meet the law's purpose). ‘Revolving doors’ practices were also identified (n = 13), such as when an ex-minister, who was later the president of the trade association AB Chile, lobbied in this latter capacity Ministers and Under-secretaries of Economy and Health regarding the front-of-package labelling regulations. There was also one example of ‘using the law to obstruct policies’ (AB Chile discussing front-of-package labelling regulations with the Minister of Economy regarding ‘intellectual property and trademark’).

**Table 3:** Action strategies used by commercial actors during lobbying activities in Chile between 2014 and 2022.

Strategies	Mechanisms	Number of meetings
Access and influence policymaking	Access policymakers and policy spaces	37
	Attempt to influence policy processes and outcomes	83
	Manage policy venues	–
Use the law to obstruct policies	Use legal challenges to policy pre- and post-adoption	1
	Use the law to undermine policy-making/public health community	–
Manufacture public support for corporate positions	Coordinate and manage industry strategies	–
	Form business alliances	15
	Secure support beyond business	7
	Fabricate allies	–
	Operate through third parties	4
	Maximise corporate–favourable media content	–
Shape evidence to manufacture doubt	Undermine and marginalise unfavourable research/information	–
	Produce or sponsor favourable research/information	16
	Amplify and blend corporate favourable evidence into public record and discourse	1
Displace and usurp public health	Undermine the rationale for statutory policies on corporate practices	3
	Deliver individual-level interventions	30
	Promote ‘harm reduction’ as public health goal	–
	Deliver education and training to public health professionals	–
	Weaken the public health community	–
Manage reputations to corporate advantage	Repair and nurture corporate reputations	115
	Discredit public health community	–

Examples of ultra-processed sugary food and drink industries attempting to ‘manufacture public support’ were also identified. Industries formed alliances to strengthen their influence by creating the impression of a broad consensus. In 41 meetings, representatives from different industries worked together (e.g., meetings requested by AB Chile lobbyists also included representatives from Nestle and the Chilean Federation of Industries (SOFOFA)). Business-related associations, such as those involved in commerce and advertising, also lobbied with similar arguments (e.g., the National Advertising Association proposing a new ‘healthy message’ because the one created by the Ministry of Health—‘Prefer foods with fewer warning labels, better if they don't have them’–was not considered appropriate because, in their opinion, it did not comply with the intention of the law). Moreover, larger industries were supported by institutions outside of business such as the Italian Embassy lobbying for Ferrero regarding the ‘effects of the food labelling regulations’. Four examples of ‘operating through third parties’ were also observed in relation to PepsiCo. Without having lobbyists registered in their name, the company was mentioned in four meetings by representatives of other associations when discussing social responsibility programmes in which they were involved (e.g., a non- PepsiCo-affiliated ‘private interests’ manager presenting a programme established together with PepsiCo to support small business owners).

Nurturing corporate reputation was also a commonly identified mechanism. Industries frequently met to ‘manage reputations to corporate advantage’. This referred to highlighting their corporate social responsibility and support to local communities, and seeking associations with public institutions (e.g., Coca-Cola presenting their community programmes to support small neighbourhood shops to the Minister of Economy and the Under-secretaries of Social Development and Treasury). Moreover, in two meetings the industry commitment to public health was highlighted while distancing themselves from the practices of others that did not follow the same approach (e.g., trade association Chilealimentos not sharing ‘AB Chile's approach to the front-of-package labelling regulations' which included media campaigns to discredit the regulations).

There were also 33 examples of actions to ‘displace and usurp public health’. Meetings were used as opportunities to advocate for downstream individual-level interventions (e.g., Chilealimentos seeking to partner with the Ministry of Social Development to extend their healthy ‘lifestyles’ educational programme) or industry-friendly policies (e.g., Coca-Cola discussing their innovation efforts to provide healthier alternatives to consumers). In addition, lobbying was used to directly provide authorities with information favourable to industry interests. For example, the food company Carozzi met with the Ministry of Health to show a study on the public opinion of the front-of-package labelling regulations and propose modifications to ‘improve it’.

### Objectives

A diverse range of short- and long-term objectives were identified (Table 4). Examples of short-term objectives targeting specific public health policies as defined by the model include commercial actors aiming to shape (e.g., trade association AB Chile voicing their disagreement with adding a new category of sugary drinks to the sugar-sweetened beverages tax), undermine (e.g., Nestle requesting discussions related to deficiencies in the interpretation of healthy messages of their imported products by the Regional Health Authority) or amend (e.g., trade association Chilealimentos proposing to ‘strengthen’ the front-of-package labelling regulations) policies in industry-friendly ways during their formulation and implementation stages, or presenting them as ineffective (e.g., AB Chile stating that the front-of-package labelling regulations had not been an effective public policy).

**Table 4:** Objectives of lobbying by commercial actors during in Chile between 2014 and 2022.

**(1) Short-term objectives**	**Number of meetings**
Suppress public health concerns and keep them out of public discourse (agenda setting)	–
Contribute to shaping policy (formulation)	13
Block, substitute or amend policy to weaken it (deliberation/adoption)	13
Sabotage policy by preventing, delaying, undermining or reversing it (implementation)	22
Present policy as failed and ineffective so it will not be implemented elsewhere (evaluation)	3
Not classified	38
**(2) Long-term objectives**	**Number of meetings**
Normalise industry as a policy partner	65
Disenfranchise the public health community	–
Re-configure and constrain public health as a non-regulatory, individual-focused practice in which corporations are essential	41
Denormalise precautionary approaches to narrow policy scope	–
Re-configure scientific standards to make it more difficult to demonstrate both the need for and effectiveness of policy.	5
Build rapport with authorities^a^	60
Not classified	7

a Objective added to the original Corporate Political Activity Model.^13^

Examples of long-term objectives aiming to normalise these industries as policy partners (e.g., proposing public-private collaborations or increasing their participation in public advisory boards), lobbying for downstream individual-level interventions (e.g., Nestle presenting their ‘Healthy Children’ educational programme and requesting to work together with governmental institutions) which can shift the focus away from upstream strategies, and influence scientific standards to be industry-friendly (e.g., Chilealimentos requesting participation in Codex Alimentarius meetings to call for ‘sensible regulations based on science which do not create barriers to trade’) were also identified.

A new long-term objective was added (i.e., ‘build rapport with authorities’) to classify meetings where the aim was to broadly strengthen relationships with authorities without targeting specific public health policies (e.g., ‘greetings’ from AB Chile to the new Minister of Economy).

There were nine examples of industries targeting both short- and long-term objectives in the same meeting. For example, Nestle proposed modifications to the front-of-package labelling regulations (short-term) and requested a public-private partnership with the Ministry of Health (long-term) in the same meeting. Forty-five meetings could only be classified within the broader objective type due to lack of detailed information in the register.

## Discussion

Using the public Lobby Registry in Chile, this study showed that ultra-processed sugary food and drinks industries used extensive lobbying practices, including meeting with high-ranking government officials and Ministers and employing a number of different strategies to influence public policies according to their interests. Several examples demonstrate how industries present themselves as ‘good actors’ and public health policies as ‘bad solutions’ in an attempt to steer the emphasis towards downstream individual interventions, influence policymaking, and foster corporate reputations.

The findings corroborate previous studies on corporate political activities in Chile^15^ and globally.^11^^,^^16^^,^^27^ The present study expanded the claims and actions identified by Mialon and collegues^17^ by using the recently developed Corporate Political Activity model^13^ to explore lobbying activities in Chile. The model was further enriched by new context-specific examples for claims, actions, and objectives. Furthermore, a new claim category (‘policy is ill-thought-out’), and a new long-term objective (‘build rapport with authorities’) were added based on the records studied. Lobbying activities to build rapport with authorities have also been identified by previous studies,^16^^,^^17^ but not classified as a long-term industry objective. Conversely, the new claim category and new long-term objective identified here could be due to employing the new model that allowed for the identification of more subtle framing strategies used by commercial entities or also due to the narrower focus of this study. It is also possible that these are only applicable in the local lobby context. However, this is unlikely due to their broader nature that facilitates wider applicability. The new claim category rather than directly criticising the front-of-package labelling regulations, queried its implementation, thereby setting the context for claims about policy inconsistencies and lack of clarity. This contributed to the policy being discredited particularly when the same actors also embarked on other more direct challenges to the regulations. Similarly, the new long-term objective added (‘build rapport with authorities’) may be seen as good-natured. However, it can also be the first step to facilitate future more direct opposition to public health policies.

No examples of the framing categories ‘the trivial and individual problem’ and ‘the ‘bad’ actors' were observed. This study used a model intended to capture a wide range of political activities but focused on only one aspect of political activity (i.e., lobbying). Thus, not all strategies included in the model were expected to be observed as not all corporate political activities may be suitable for each of the model strategies. Additionally, lobbying tended to use a ‘friendly’ approach even when criticising policies overall. Few examples of openly hostile meetings were observed even though aggressive strategies have been identified in other studies in Chile^15^ and worldwide.^11^^,^^16^^,^^27^ A ‘friendly’ approach to lobbying is within the long-term interests of industries because it can be perceived as constructive and facilitates building strong relationships with governments to influence policymaking. Furthermore, lobbying activities often targeted multiple objectives within the same meeting in order to make the most use of these opportunities to engage with authorities. There were also several examples of this ‘friendly’ approach with industries and related interest groups promoting nutritional education programmes and other social strategies, even when such programmes were already provided by public services.

Providing advice and proposing modifications to existing regulations was another relatively common activity (14 records) which, in most cases, was not requested by government authorities, and was carried out without following existing official routes. More importantly, such advice tended to target evidence-based policies with non-evidence-based proposals (e.g., Carozzi presenting a methodologically unsound proposal according to Ministry of Health representatives to ‘improve’ the front-of-package labelling regulations).

The inappropriate lobbying practices identified in this study took place in Chile within a strong neoliberal political context, which favours political and economic norms that increase the power of industry actors and facilitate the misuse of long-established relationships between public and private actors.^11^^,^^17^ This has led to a strong impact of lobbying on policymaking at the national level by, for example, delaying the implementation of the front-of-package labelling regulations.^17^ Thus, being aware of the context-specific characteristics of lobbying and how it may influence new regulations from the initial proposal up to their final form is fundamental for successful public health policymaking. Often, commercial actors are enabled by governments and other public organisations that should hold them to account as a result of the power exerted by these same companies to influence norms, ideas and beliefs in their own interest. This leads to industry-friendly environments that shape policy proposals from their inception.^11^ In this context, the conceptualisation of power sheds light on why and how seemingly innocuous meetings can lead to such strong influence over policymakers. According to Fuchs and Lukes, there are three faces to power: instrumental (ability to influence other actors), structural (ability to use material conditions to shape the structures where actors interact), and discursive (ability to influence processes through shaping norms and values).^11^ For example, political economy research in Chile has observed that companies with high instrumental and structural power have successfully shaped policy outcomes through lobbying (e.g., a tax reform that included the sugar-sweetened beverage tax in 2014).^28^

When lobbying to oppose public health policies, ultra-processed food industries tend to use similar claims and mechanisms.^13^ This has implications for policymaking as it allows bespoke counterarguments and actions to be formulated. One essential step is to strengthen mechanisms to regulate lobbying. In Chile, the law on lobbying has increased transparency and improved its management. However, no formal mechanisms exist to regulate inappropriate lobbying. This is the case in several countries where management and transparency of corporate political activities has improved but effective regulatory mechanisms are still lacking.^29^ In this sense, the WHO Framework Convention on Tobacco Control provides good examples of how to limit industry influence in public policy decisions^30^ for example, by ranking countries based on their ‘independence’ from the tobacco industry.^31^

This study has some limitations. First, the quality of the data varies as responsibility for accuracy lies with each government institution. Clear guidance and better harmonisation of record keeping is needed to better safeguard lobbying. Second, the records provide only a brief summary written by public servants. This did not allow to directly analyse the discourse used by commercial actors. Third, the data collected only include official meetings and do not consider informal meetings and other ‘closed door’ practices that may be used by the industry. Fourth, the definition of the food industry actors and related commercial actors was not straightforward as some commercial actors may have a wide range of products and participate in diverse aspects of commerce which hinder simple categorisation.^32^ Because of this, it is unlikely that all food-industry-related commercial actors were identified as some may not be traditionally associated with commercial activities. However, searching both lobby websites using the name of the commercial actors and then affiliations of registered lobbyists partly addressed this limitation as it allowed for the identification of the most relevant actors lobbying in Chile. During the period studied, Chile experienced major changes in the sociopolitical context including the COVID-19 pandemic, and social protests that could have influenced the frequency of meetings or topics discussed. It is not possible to assess from the records whether these affected lobbying activities. However, this is not directly related to the aim of this study regarding the description of lobbying activities based on existing records.

This study provided a detailed overview of lobbying characteristics in Chile by ultra-processed sugary food and drinks industries and related commercial actors during an extended period in which major public health policies were being implemented, providing a closer look at corporate political activities in the Latin American region. By focusing on ultra-processed sugary food and drink industries and related corporate interest groups, this study provides a nuanced view of this particular group, their strategies to influence public health and provide insights on how to manage them. This can be of relevance for policymakers, advocates and researchers in other Latin American countries with similar characteristics going through similar processes, for those working on conditions caused by excessive sugar consumption, or as a case study of interest for political science research.

Lobbying was a corporate political activity extensively used during a period when major public health policies were being discussed and implemented in Chile. Lobbying aimed to nurture business reputations and influence policymaking by presenting themselves as supporters of health while at the same time criticising or querying evidence-based upstream public health policies adverse to industry interests. Lobbying strategies varied to meet industry objectives and targeted a diverse range of government institutions including high-ranking officials. Understanding lobbying practices in their different contexts, particularly in low-middle income countries where lobbying takes a bolder shape, calls for strong countermeasures such as tightening regulations to hinder inappropriate influence in public health policy making and allowing industry input only through the standard processes and routes that call for evidence-based arguments and apply to all stakeholders, with the aim of to creating healthier food systems and societies.

## Contributors

All authors conceived and contributed to conceptualise the study. YA designed, extracted, and led analysis of the data. YA translated data from Spanish to English and all authors had full access to all data in the study (in English). GT, RW, AH, and TB provided supervision and contributed to the analysis of data and interpretation of results. YA drafted the manuscript. GT, RW, AH, and TB reviewed and edited the manuscript. All authors approved the final draft and accepted responsibility to submit for publication.

## Data sharing statement

Meeting records are publicly available in the following two Chilean websites: the Chilean Platform for Lobbying (https://www.leylobby.gob.cl/) and ‘InfoLobby’ (https://www.infolobby.cl/) in Spanish.

Supplementary material includes meeting codes (when available) and data extracted from meeting records translated into English by YA (native Spanish speaker).

## Declaration of interests

All authors declare no competing interests. This study will be part of a PhD thesis. YA's PhD studies are funded by ANID (Agencia Nacional de Investigación y Desarrollo—Chile)—Scholarship ID 72210219 while funding to cover article publication fees is provided by UCL. None of those institutions had any influence over the research undertaken. GT declares annual honoraria unrelated to this research as an associate editor of the Community Dentistry and Oral Epidemiology Journal and Chair of the Platform for Better Oral Health in Europe (2019–2023).

## Appendix A Supplementary data

Supplementary Tables

Abstract in Spanish
