# Supplementary material for: Analysis of public records of lobbying practices of the ultra-processed sugary food and drink industries in Chile: a qualitative study
Source: Lancet Reg Health Am. 2024 Jun 9;35:100794. doi: 10.1016/j.lana.2024.100794 (PMC11294830; doi:10.1016/j.lana.2024.100794)
Supplement: Supplementary Tables [file mmc1.pdf]

Analysis of lobbying practices of the ultra-processed sugary food and drink industries in Chile: a qualitative study

Supplementary Material

Table of contents.

1. Supplementary Methods: Keywords used in Lobby Registry websites search……...Page 2-3
2. Supplementary Table 1. Data extracted from Lobby Registry records…….……Page 4-22

1. Supplementary Methods: Keywords used in Lobby Registry websites search

Major Food and drinks industry actors in Chile

- Coca Cola
- Nestlé
- Ferrero
- Mars
- Bimbo
- Carozzi
- Soprole
- Andina
- Embonor
- General Mills
- Mondelez
- PF Alimentos
- Naturell
- Kellog's
- Danone
- PepsiCo

Related corporate interest groups[1]

Type	Name
General Food Industry	• AB Chile<br>• Chilealimentos
Primary production, processing and ingredients	None identified
Food manufacturing and retail	• Chilean supermarket union association
Branding and advertising	• National Advertisers association (ANDA)
General business and trade	• Santiago Chamber of Commerce<br>• Federation of Chilean Industries (SOFOFA)
Research and science communication	• Asociación chilena de ciencia y tecnología de alimentos – SOCHITAL (Chilean Association of Food Science and Technology)<br>• Instituto de Nutrición y Tecnología de los Alimentos – INTA (Institute of Nutrition and Food Technology)<br>• Sociedad Chilena de Nutrición – SOCHINUT (Chilean Nutrition Society)<br>• Asociación Chilena de Nutrición Clínica Obesidad y Metabolismo – ACHINUMET (Chilean Association of Clinical Nutrition Obesity and Metabolism)<br>• Colegio de nutricionistas (School of Nutritionists)<br>• Sociedad Chilena de Endocrinología y Diabetes – SOCHED (Chilean Society of Endocrinology and Diabetes)<br>• Sociedad Chilena de Obesidad – SOCHOB (Chilean Obesity Society)<br>• International Life Sciences Institute (ILSI) Sur Andino.
Lobbying, legal and public relations	None identified
Sustainability/Corporate social responsibility/multistakeholder initiatives	None identified
Specialised nutrition and baby food	None identified
Others	• Italian embassy

[1]Based on Slater et al. 2024 (https://doi.org/10.1186/s12992-024-01020-4)

Policymaking-related words*

- Etiquetado de alimentos (Food labelling)
- 20.606 (food-labelling law)
- Ley de etiquetado (labelling law)
- Saludable (healthy)
- Alimentación (feeding)
- Obesidad (obesity)
- Publicidad de alimentos (food advertising)
- 20.869 (advertising regulations law)
- Nutricion (nutrition)

* English translation provided in brackets for original words used in Spanish.

2. Supplementary Table 1. Data extracted from Lobby Registry records

Industry actor	Government body	Year	Role of civil servant	Topics discussed	Representative characteristics	Code
AB Chile (Association of Food and Drinks Industries of Chile)	Ministry of Economy	2015	Under-secretary of Economy	Implementation law 20,606 and food legislation	4 Ab Chile, 1 nestle, 1 CCU	AH001AW0061467
AB Chile (Association of Food and Drinks Industries of Chile)	Ministry of Economy	2015	Minister	Food labelling	1 AB Chile, 1 SOFOFA, 1 no info	AH001AW0063031
AB Chile (Association of Food and Drinks Industries of Chile)	Ministry of Foreign Affairs	2015	Head of General Directorate of International Economic Relations	Implementation law 20,606 and food legislation	4 AB Chile, 1 Nestle-AB Chile, 1 CCU-AB Chile	AC002AW0061475
AB Chile (Association of Food and Drinks Industries of Chile)	Ministry of Health	2016	Minister	Food labelling	3 SOFOFA	No Code (20 Apr 2016 12:00)
AB Chile (Association of Food and Drinks Industries of Chile)	Ministry of Health	2016	Head of the Food and Nutrition Department (DIPOL)	Questions about implementation decree of law 20,606, particularly on advertising targeting children. The Ministry explains the regulations and give examples.	2 AB Chile - SOFOFA	No Code (22 Dic 2016 20:30)
AB Chile (Association of Food and Drinks Industries of Chile)	Ministry of Health	2016	Under-secretary of Public Health	Implementation of food labelling law. They declare that beyond technical differences regarding the implementation decree process, they offer their full collaboration and support to the Ministry of Health. The Ministry thanks their support. AB Chile highlights two concerns: if the implementation date impacts production and commercialisation or elaboration. The Ministry replies that it is a judicial matter considering they had one year to prepare. They also request to consider transition dates to change warning labels considering the stages of implementation. The Ministry can discuss this in the future.	5 AB Chile, 1 Ab Chile-nestle	No Code (22 Jan 2016 12:00)
AB Chile (Association of Food and Drinks Industries of Chile)	Ministry of Health	2016	Minister	Report on evaluation of implementation decree of law 20,606	1 SOFOFA, 2 Ab Chile-SOFOFA, 1 SOFOFA - supermarket association	No Code (28 Dic 2016 12:00)
AB Chile (Association of Food and Drinks Industries of Chile)	Presidency	2016	Minister Secretary General of the Presidency	Food labelling	1 SOFOFA, 1 AB Chile-SOFOFA	AF001AW0069792
AB Chile (Association of Food and Drinks Industries of Chile)	Ministry of Economy	2017	Minister	Implementation decree food labelling law, intellectual property and trademark	2 AB Chile	AH001AW0234730
AB Chile (Association of Food and Drinks Industries of Chile)	Ministry of Health	2017	Under-secretary of Public Health	Compliance with the food labelling regulations on labelling of infant formulas. They have reduced added sugars to their infant formulas to be below the thresholds of the law 20,606. What happens with other infant formulas? Other companies are above the thresholds and do not have warning labels and make advertising targeting children	3 Nestle	No Code (16 Jun 2017 15:00)
AB Chile (Association of Food and Drinks Industries of Chile)	Ministry of Health	2017	Under-secretary of Public Health	Questions regarding labelling of chocolates. The Ministry asks about the chocolate production process and the mix of ingredients. SOFOFA will send a document with information and a proposal on the topic. MINSAL will evaluate the proposal and will give an answer	1 AB Chile - SOFOFA	No Code (27 Apr 2017 9:30)
AB Chile (Association of Food and Drinks Industries of Chile)	Ministry of Economy	2018	Minister	Greetings to new authorities	2 AB Chile	AH001AW0445549

AB Chile (Association of Food and Drinks Industries of Chile)	Ministry of Health	2018	Head of the Food and Nutrition Department (DIPOL)	Greetings to new authorities in the Ministry. Show them a study on food labelling policy by CADEM (private company) for Carozzi (a big national food industry) and another study by ABChile regarding the mandatory healthy message in advertising.	2 AB Chile	AO001AW0426863
AB Chile (Association of Food and Drinks Industries of Chile)	Ministry of Health	2018	Under-secretary of Public Health	Greetings to new authorities in the Ministry. Ask why is taking so long to include the private sector in the discussion of public policies. "AB Chile and its associated industries are available for anything the Ministry needs". The industry has interest in supporting health public policies. Talk regarding food labelling. They have observed some signs that the food labelling has not been an effective public policy.	2 AB Chile	AO001AW0426864
AB Chile (Association of Food and Drinks Industries of Chile)	Ministry of Health	2018	Head of the Division of Healthy Public Policies and Promotion (DIPOL)	Proposal to modify the decree that regulates the mandatory healthy message in food advertising. Request of a public-private work group to define healthy messages.	1 Nestle, 2 AB Chile	AO001AW0486671
AB Chile (Association of Food and Drinks Industries of Chile)	Ministry of Social Development	2018	Head of Elige Vivir Sano (Choose living healthy)	Food labelling law	2 AB Chile, 1 Nestle-AB Chile	AI008AW0486670
AB Chile (Association of Food and Drinks Industries of Chile)	Ministry of Health	2019	Head of the Food and Nutrition Department (DIPOL)	Request to speak with Minister of Health. How are portions measured. Concept of variability. Concept of single pack. Requests to know the views of the Ministry regarding homemade measures and portions. The department will analyse this with their technical team	2 AB Chile	AO001AW0707014
AB Chile (Association of Food and Drinks Industries of Chile)	Constitutional Convention	2021	Member of the constitutional convention	Food and the constitution	4 AB Chile, 2 SOFOFA	NR009AW1047194
AB Chile (Association of Food and Drinks Industries of Chile)	Constitutional Convention	2021	Member of the constitutional convention	right to food	2 AB Chile	NR009AW1050590
AB Chile (Association of Food and Drinks Industries of Chile)	Ministry of Health	2021	Head of the Food and Nutrition Department (DIPOL)	To analyse the change of criteria regarding nectars to be considered non-alcoholic beverages (taxable)	3 AB Chile	AO001AW0976835
AB Chile (Association of Food and Drinks Industries of Chile)	Ministry of Health	2021	Head of the Food and Nutrition Department (DIPOL)	To discuss their disagreement regarding the tax introduced by the Internal Revenue Service to nectars and non-alcoholic beverages due to changes in the Sanitary Regulation for Food products.	4 AB Chile	AO001AW1006555
AB Chile (Association of Food and Drinks Industries of Chile)	Tax Office	2021	Head of Service	Request for an audience regarding the process of regulatory modification through a circular called "Application of additional tax on nectars, beverages isotonic and hypotonic drinks, established in letter a) of article 42 of Decree Law No. 825 of 1974 carried out by the tax office. The service informs the following: Their views are heard, and queries answered.	3 AB Chile	AE006AW0988585
AB Chile (Association of Food and Drinks Industries of Chile)	Treasury	2021	Civil servant - tax policy coordinator	Implementation of additional tax to nectars, isotonic drinks and drinks	6 AB Chile, 1 SOFOFA	AE001AW1004460
AB Chile (Association of Food and Drinks Industries of Chile)	Constitutional Convention	2022	Member of the constitutional convention	right to food	2 AB Chile	NR009AW1060844
AB Chile (Association of Food and Drinks Industries of Chile)	Constitutional Convention	2022	Member of the constitutional convention	To present two major concerns such as the Freedom of Entrepreneurship and Economic Public Order. They also state that they are following up on the "Food Sovereignty" Initiative, which is being analysed by the Environment Commission.	2 AB Chile	NR009AW1064462
AB Chile (Association of Food and Drinks Industries of Chile)	Ministry of Economy	2022	Head of Investment and promotion of industries	To introduce themselves, their work and topics of interest	2 AB Chile	No Code (1 Jun 2022 10:00)
AB Chile (Association of Food and Drinks Industries of Chile)	Ministry of Foreign Affairs	2022	General director of bilateral economic affairs	To inform their interest in the technical protocols of the Pacific Alliance. To discuss about non-notified technical manuals to be present in open consultation at WTO	2 AB Chile	AC007AW1092373

AB Chile (Association of Food and Drinks Industries of Chile)	Ministry of Health	2022	Head of the Food and Nutrition Department (DIPOL)	To present AB Chile, their work and topics of interest. To greet new authorities and present the new AB Chile president. They proposed 2 studies: food lost in processing plants (because of FAO sensibilisation) and international deficiencies for older adults. MINSAL informs they are participating together with SENAMA (National service for older adults) in the second study and that is of great importance for them. The department will make a proposal regarding the first study	2 AB Chile	AO001AW1110078
AB Chile (Association of Food and Drinks Industries of Chile)	Ministry of Health	2022	Head of the Food and Nutrition Department (DIPOL)	To present a project for older adults (nutritional deficiencies). They mention that they participate in some advisory boards supporting public policies. Request a technical committee for INTA to inform of their progress. Also mention a agreement with FAO to look for topics of common interest to make new projects.	2 AB Chile	AO001AW1238485
AB Chile (Association of Food and Drinks Industries of Chile)	National Service for older adults	2022	Head of Service	To show their project for older adults and ask for future meetings to continue discussing the project	2 AB Chile	AI007AW1238511
AB Chile (Association of Food and Drinks Industries of Chile)	Treasury	2022	Under-secretary of Treasury	observatory of basic food basket and food contingency	2 AB Chile, 1 no info	AE001AW1117670
AB Chile (Association of Food and Drinks Industries of Chile)	Ministry of Health	2015	Under-secretary of Public Heath	Food labelling law	6 AB Chile	No Code (17 Dic 2015 15:00)
ARCOR (confectionery)	Ministry of Sports	2016	Under-secretary of sports	To present their work in schools (programme 'moving schools'). To look at potential alliances and work together with the Ministry and their programme 'Holistic Sport Schools'. They think that they share the same objectives with the Ministry such as promoting physical activity since childhood.	1 Arcor Chile foundation for equal opportunities in childhood.	BA001AW0189439
ARCOR (confectionery)	Integra Foundation (network of nurseries and kindergartens)	2018	Executive Director	Update of the Agreement for the Training Programme for instructors in active life	1 Arcor Chile foundation for equal opportunities in childhood.	FU001AW0540855
ARCOR (confectionery)	Ministry of Education	2018	Under-secretary of preschool education	To present the work of their foundation in different municipalities of the Metropolitan region. They request sponsorship for a conference called "Moving for a healthy childhood"	1 Arcor Chile foundation for equal opportunities in childhood.	AJ014AW0465813
ARCOR (confectionery)	Ministry of Sports	2018	Head of political division and sports management	To contribute to a public-private alliance to promote active and healthy lifestyles in schools. To contribute to the visibility and importance in public policies of healthy lifestyles	1 Arcor Chile foundation for equal opportunities in childhood.	BA001AW0465857
ARCOR (confectionery)	Ministry of Education	2019	Under-secretary of preschool education	The secretary cannot be present, the chief of staff meets ARCOR representatives. Representatives inform the launch of a study for alternative school modalities. They also look for sponsorship from the Ministry.	1 Arcor Chile foundation for equal opportunities in childhood.	AJ014AW0631734
ARCOR (confectionery)	Ministry of Education	2019	Chief of Staff Secretary of Childhood	To request sponsorship and presence of the Secretary of Childhood in an annual conference "Moving for a healthy childhood" developed together with their foundation and 2 private universities	1 Arcor Chile foundation for equal opportunities in childhood.	AI009AW0693584
ARCOR (confectionery)	Ministry of Sports	2019	Head of political division and sports management	To introduce their Foundation and their work to promote active and healthy lifestyles in children at schools in the Metropolitan region. Request sponsorship for an international conference about promotion of physical activity in childhood	1 Arcor Chile foundation for equal opportunities in childhood.	BA001AW0657497

Carozzi (confectionery, juices)	Ministry of Economy	2018	Minister	The objective is to provide information on the implementation of the Food Labelling Law regulations, which are limiting free trade, hindering national exports and preventing the use of brands and their commercial properties, seriously detrimental to private investment.	2 Carozzi	AH001AW0429324
Carozzi (confectionery, juices)	Ministry of Foreign Affairs	2018	Head of General Directorate of International Economic Relations	The need for joint work between the public and private sectors to move towards common food labelling at the international level that, along with promoting informed eating, contributes to international integration and cooperation, and helps reduce barriers to free trade.	2 Carozzi, 1 No info	AC002AW0504029
Carozzi (confectionery, juices)	Ministry of health	2018	Minister	Proposal to modify food labelling (add more info to it). Proposal to contribute from the private sector to public policies targeting obesity.	2 Carozzi	AO001AW0575659
Carozzi (confectionery, juices)	Ministry of health	2018	Head of the Division of Healthy Public Policies and Promotion - DIPOL	Carozzi asks for a meeting to share their views on current public policies being implemented in the country to tackle obesity and to present studies about the food labelling regulations in order to contribute from the industry to improve this public policy and to correct some inconsistencies of great health relevance.<br>From the Ministry of Health summary: Carozzi gives their observations on the mandatory healthy message for advertising that they think may have inconsistencies with the public policy. Propose review of food profiles, either by portions or every 100 grams. Mention they are developing an app to educate people on the food labelling. Discuss the dates for the 3rd threshold implementation (ask to consider elaboration date, not selling date). Ask about news on food taxation. Ministry of Health explains intersectoral approach to tackle obesity through the 'Chose living healthy' programme (they suggest Carozzi talking directly with the person in charge of the programme). Discussion on implementation on law 20,606: Ministry is constantly monitoring and evaluating the process and will share with the public all the studies on it when they are done.	2 Carozzi	AO001AW0426751
Carozzi (confectionery, juices)	Ministry of Social Development	2018	Head of Elige Vivir Sano (Choose living healthy)	Carozzi raises the need to implement an obesity discussion table. Commitments/Agreements, Elige Vivir Sano states that work is being done to implement a plan against obesity	2 Carozzi	AI008AW0576126
Carozzi (confectionery, juices)	Ministry of Social Development	2018	Head of Elige Vivir Sano (Choose living healthy)	To request to work together. Commitment to support projects that promote healthy lifestyles and request sponsorship for the "Carozzi Olympics"	2 Carozzi	AI008AW0472890
Carozzi (confectionery, juices)	Ministry of Social Development	2018	Head of Elige Vivir Sano (Choose living healthy)	To discuss the food labelling law. The service explains their goals for the year and that they can sponsor industry programmes that promote healthy lifestyles of Chileans but not foods specifically. They agree to continue discussing ways to clearly inform consumers of which foods they should prefer.	2 Carozzi	AI008AW0448481
Carozzi (confectionery, juices)	Ministry of Sports	2018	Minister	Proposal to contribute to public policies that give more and better information to people and promote physical activity.	2 Carozzi	BA001AW0576119
Carozzi (confectionery, juices)	Municipality of Vina del Mar	2018	Community development director	To get closer to the Municipality and their plans with the community near Carozzi plant. To plan activities to promote healthy lifestyles and identify Neighbourhood councils. The Municipality has already worked with Carozzi in specific activities such as sports events. Carozzi has two programmes. to visit schools and to take them for guided tours to their plant. They also have activities focusing on pets.	2 Carozzi	No Code (25 Oct 2018 17:00)

Carozzi (confectionery, juices)	National Consumer Service	2018	National Director	Animal welfare program and implementation at points of contact with the consumer.	2 Carozzi	AH009AW0534987
Carozzi (confectionery, juices)	Treasury	2018	Under-secretary of Tresuary	Impact of regulations on the food industry, especially in food categories with low consumption rates in the population	2 Carozzi	AE001AW0463182
Carozzi (confectionery, juices)	Ministry of health	2019	Head of the Food and Nutrition Department (DIPOL)	Proposal to improve food labelling and to show study about public opinion of the proposal. From the Ministry of Health: interest in listening to proposals to improve the law. Identified important methodological issues in study showed. The Department of Nutrition and Foods highlights the error in the phrase that "the law has not had good results when looking the numbers regarding obesity", the department clarify that the main objective of the law is not that one. Carozzi is referred to look at the Ministry of Health website to learn about the law 20,606.	2 Carozzi	AO001AW0682425
Carozzi (confectionery, juices)	Ministry of Social Development	2019	Minister	Proposal to contribute to public policies through providing more and better information to people to promote healthy habits. To present the work of the company in improving their foods (17 categories).	2 Carozzi	AI007AW0580445
Carozzi (confectionery, juices)	Ministry of Social Development	2019	Secretary of Elige Vivir Sano (Choose living healthy)	Present a study and a proposal for warning labels	2 Carozzi	AI008AW0641538
Carozzi (confectionery, juices)	National Consumer Service	2020	Deputy director of economic studies and education	App development "Comamos informados" (Let's eat informed). Its objective is to provide more and better information to consumers. App is brandless, any company can join . The app gives recommendations tailored according to weight and height of individuals according to FAO and WHO recommendations. SERNAC reiterates the existence of a platform with similar purpose (Mi codigo verde - My green code) and the necessity for foods to have warning signs. SERNAC suggest Carozzi to talk to the Ministry of Health about their app.	1 Carozzi	AH009AW0878824
Carozzi (confectionery, juices)	Ministry of Social Development	2021	Secretary of Elige Vivir Sano (Choose Living Healthy)	To present an app (Comamos Informados - let´s eat informed) developed by Carozzi that aims to provide clear and simple nutritional information and to contribute to an informed and conscious consumption of foods.	1 Carozzi	AI008AW0911265
Carozzi (confectionery, juices)	Municipality of San Bernardo	2021	Mayor	To present people in charge of the Carozzi company. To show the benefits given by the company to employees and the community	1 Carozzi	MU281AW0861572
Carozzi (confectionery, juices)	Municipality of San Bernardo	2021	Mayor	To present the company (has 50% women workers). Gives land belonging to the company to build a rural health service (the land is already being assessed by the Ministry of Social Development). Mayor discusses aspects related to the land and access to main roads. The company mentions their work with the community, they want to install recycling points, their permanent support to a local neighbour and giving foods to communal cooking pots. The mayor is looking for companies to be more participative in the welfare of the communities of his municipality. Company asks for help in recycling projects and to share job offers through the municipality.	4 Carozzi	MU281AW0968249
Carozzi (confectionery, juices)	Municipality of Vina del Mar	2021	Mayor	Greetings to new Mayor. Present their work with the community and their sustainibility plans. Offer an alliance to implement new programmes and help with public policies in the municipality.	2 Carozzi	MU340AW0968445
Carozzi (confectionery, juices)	National School Aid and Scholarship Board	2022	Head of service	Carozzi wants to improve their products used by JUNAEB in the school feeding programme in quality and availability. Offers JUNAEB support to resolve problems they may have based on their innovation, knowledge and understanding of the global landscape. Offer training programmes to food	1 Carozzi	AJ009AW1113041

				manipulators. JUNAEB highlights the importance of transparency in public-private partnerships, so all their offers have to be studied to ensure it meets national regulations. JUNAEB requests Carozzi to send their proposal by email to analyse it in more depth.		
Carozzi (confectionery, juices)	Municipality of Teno	2023	Mayor	To discuss sport activities in Teno	1 Carozzi, 2 no info	MU316AW1267816
CCU (United Brewery Company) -Second largest soft drink producer	Municipality of Quilicura	2016	Mayor	Diagnostic study of the Municipality to implement a model of relationship between the community and CCU.	2 CCU	MU250AW0139611
CCU (United Brewery Company) -Second largest soft drink producer	Municipality of Temuco	2016	Civil servant	Interview about the social role of companies and their perception as part of CCU community relations plan.	1 CCU, 1 Temuco Municipality	MU315AW0145606
CCU (United Brewery Company) -Second largest soft drink producer	Municipality of Temuco	2016	Councillor (local authority)	Interview about the social role of companies and their perception as part of CCU community relations plan.	2 CCU	MU315AW0145614
CCU (United Brewery Company) -Second largest soft drink producer	Municipality of Temuco	2016	Councillor (local authority)	Interview about the social role of companies and their perception as part of CCU community relations plan.	2 CCU	MU315AW0145620
CCU (United Brewery Company) -Second largest soft drink producer	Ministry of Environment	2017	Minister	To invite the Minister to participate of their recycling campaign and the telethon task '27 hrs of love CCU 2017'. During the meeting the joint work between the Ministry and CCU regarding the recycling campaign for Telethon was discussed. CCU requested support from the Ministry to make the largest campaign to date.	1 CCU	AW002AW0301174
CCU (United Brewery Company) -Second largest soft drink producer	Ministry of health	2017	Head of the Food and Nutrition Department (DIPOL)	Food for athletes and their inclusion on the category of foods specially made for athletes commercialised on other countries. They want to import new products and for different local authorities these are categorised differently. There is a coincidence between confectionery, supplement and food for athletes. It is suggested to request an audience with the secretary of public health to discuss the issue.	1 ECCU, 2 PFI investments	No Code (7 Jun 2017 14:00)
CCU (United Brewery Company) -Second largest soft drink producer	Municipality of Antofagasta	2017	Councillor (local authority)	Diagnostic study of the Municipality to implement the CCU community relationship model	2 CCU	No Code (28 Apr 2017 15:30)
CCU (United Brewery Company) -Second largest soft drink producer	Municipality of Antofagasta	2017	Mayor	Diagnostic study of the Municipality to implement the CCU community relationship model	2 CCU	No Code (4 May 2017 12:30)
CCU (United Brewery Company) -Second largest soft drink producer	Municipality of Coinco	2017	Councillor (local authority)	Changes in Cachantún (bottled water brand) plant. Show their work with the community and the plant. Inform of a study to gather information from local stakeholders through interviews. To inform of the new community programme they will do (Educar en Familia 2.0 - Educate in Family). How they will emphasise sports, education, and environment.	2 CCU	MU054AW0292643
CCU (United Brewery Company) -Second largest soft drink producer	Municipality of Los Angeles	2017	Mayor	Show the mayor their local community relations team and the characteristics of their new distribution plant in Los Ángeles.	3 CCU	MU153AW0371698
CCU (United Brewery Company) -Second largest soft drink producer	Municipality of Renca	2017	Mayor	Work with the community	1 CCU-ECCU	MU265AW0346424

CCU (United Brewery Company) -Second largest soft drink producer	Municipality of San Bernardo	2017	Civil servant	To present the current CCU CEO and to discuss topics of interest for their operations and the community considering the many years of the company in the Municipality and their two distribution centres.	2 Transports CCU	MU281AW0263696
CCU (United Brewery Company) -Second largest soft drink producer	Ministry of Economy	2018	Civil servant	To present CCU projects and their subsidiaries in the region	1 CCU, 1 ECCU-CCU	AH001AW0526548
CCU (United Brewery Company) -Second largest soft drink producer	Ministry of Environment	2018	Civil servant - Regional Ministry of Environment (Araucania region)	Protocol visit. CCU invites to a guided tour of the Temuco Plant certified as Zero Waste.	3 CCU	AW002AW0454746
CCU (United Brewery Company) -Second largest soft drink producer	Ministry of Environment	2018	Head of Communications and Press Office	To coordinate the Telethon task '27 hrs of love'. CCU in a strategic alliance with the Ministry of Environment will do the task '27 hrs of love CCU' to collaborate with Telethon children and take care of the environment	1 CCU	AW002AW0463287
CCU (United Brewery Company) -Second largest soft drink producer	Municipality of Antofagasta	2018	Mayor	To present the new CCU community relationship plan that includes art exhibitions and an educational programme	1 CCU	No Code (2 Apr 2018 15:00)
CCU (United Brewery Company) -Second largest soft drink producer	Municipality of Renca	2018	Mayor	Contribution request for COP25 in Renca	2 CCU	MU265AW0719744
CCU (United Brewery Company) -Second largest soft drink producer	Municipality of Temuco	2018	Mayor	A team will visit the Municipality and want to meet with the Mayor to present the new complementary community relations plan that includes art exhibitions and talks on responsible alcohol consumption in the programme "Educar en Familia - Educate in family". Also to update the mayor on the projects being implemented in the Municipality and to invite him to a guided tour of their plant.	5 CCU, 3 Temuco municipality, 1 no info	MU315AW0454322
CCU (United Brewery Company) -Second largest soft drink producer	Municipality of Vilcun	2018	Mayor	To ask for a meeting with the mayor to show their new plan of relationship with the community which include doing and art exposition in the area and talks about responsible alcohol consumption (programme: Educar en familia - education in family). Also, to show the progress of the projects being done in the municipality and to invite the mayor and its team to a guided visit to Temuco plant certified as "Zero residues".	3 CCU	MU336AW0454704
CCU (United Brewery Company) -Second largest soft drink producer	National Council for Culture and the Arts	2018	Ministerial regional secretary	Presentation of CCU's New Complementary Community Relations Plan, which includes holding an Art exhibition in the area and talks on Responsible Alcohol Consumption of the Educar en Familia programme. Presentation of the progress of the projects that are being carried out in the area. Invitation to participate in a guided tour of the Temuco Plant certified as Zero Waste.	4 CCU	AV001AW0454714
CCU (United Brewery Company) -Second largest soft drink producer	Ministry of Culture	2019	Civil servant	CCU arts scholarship. Invite the Ministry to its 4th version and discuss other projects being done by CCU in the arts area	2 CCU	BC001AW0711744
CCU (United Brewery Company) -Second largest soft drink producer	Municipality of Renca	2019	Mayor	Company investments on the municipality	2 CCU	MU265AW0639885
CCU (United Brewery Company) -Second largest soft drink producer	Municipality of Renca	2019	Mayor	Sports programme 2019-2021	1 ECCU, 1 ECCU-CCU	MU265AW0602933
CCU (United Brewery Company) -Second largest soft drink producer	Municipality of Quilicura	2020	Mayor	To analyse activities for the community of Quilicura in which they could support the local government	2 United Chilean Bottling Companies (ECCU)	MU250AW0816429

CCU (United Brewery Company) -Second largest soft drink producer	Metropolitan Regional government	2021	Regional Governor	Present community-oriented programmes and projects by CCU in the Metropolitan region such as Responsible alcohol consumption programmes, small retailers development, etc. Also, present infrastructure and development projects.	2 CCU	AB081AW1003071
CCU (United Brewery Company) -Second largest soft drink producer	Municipality of San Bernardo	2021	Mayor	To present a project. To offer an alliance. Development of communities in a tri-annual programme. Talk about green areas, sports and recycling. Job offers and commitment with 2 neighbours.	4 Transports CCU	MU281AW0997718
CCU (United Brewery Company) -Second largest soft drink producer	Municipality of Purranque	2022	Mayor	relationship with the community and topics regarding advertising restrictions for small businesses	1 CCU	MU242AW1201864
CCU (United Brewery Company) -Second largest soft drink producer	Municipality of Quilicura	2022	Mayor	To inform the mayor of their plant expansion plans.	3 ECCU, 1 CCU-ECCU	MU250AW1086327
CCU (United Brewery Company) -Second largest soft drink producer	Municipality of Quilicura	2022	Mayor	Greetings to new Mayor and offer support to municipality activities	2 CCU, 1 ECCU-CCU	MU250AW1058424
Chilealimentos (Association of Food Industries)	Ministry of Health	2015	Head of the Division of Healthy Public Policies and Promotion (DIPOL)	Commitment to install healthy kiosks in schools. Invitation to the inauguration in Illapel. Implementation law 20,606 and start of monitoring. Obesity observatory but on all public policies in Chile	4 Chilealimentos	No Code (30 Dic 2015 15:00)
Chilealimentos (Association of Food Industries)	Ministry of Foreign Affairs	2015	Head of Service	To include processed foods industry into Pacific Alliance (economic and development integration group formed by Chile, Colombia, Mexico and Peru) to facilitate commerce between member countries	3 Chilealimentos	AC002AW0005612
Chilealimentos (Association of Food Industries)	Ministry of Social Development	2015	Minister	To voice their concerns regarding the increasing of NCDs caused by obesity and sedentarism. They propose to implement activities together with the Ministry of Social Development to move forward public policies on healthy lifestyles especially focused on children.	2 Chilealimentos, 1 no info	AI007AW0010251
Chilealimentos (Association of Food Industries)	Ministry of Health	2016	Head of the Food and Nutrition Department (DIPOL)	Chilealimentos position regarding the law 20,606 and proposals. Chilealimentos support the Ministry of Health strategies including this law. Do not share AB Chile reaction to the law. More public policies are needed. They propose a board to for health promotion. They are aware of illegal selling of products inside schools, this is because of the limited amount of products that can be sold inside now.	3 Chilealimentos	No Code (28 Dic 2016 18:00)
Chilealimentos (Association of Food Industries)	Ministry of Health	2016	Under-secretary of Public Health	Chilealimentos represents La Feté Chocolates. They want to discuss the regulations of the law 20,606	2 Chilealimentos-Chocolates del mundo, 1 Chocolates del mundo	No Code (10 Nov 2016 14:18)
Chilealimentos (Association of Food Industries)	Ministry of Health	2016	Head of the Food and Nutrition Department (DIPOL)	To clarify doubts regarding the request by Aramark restaurants on selling non-packaged foods, particularly ready meals in schools. The ministry explains the law 20,606 and its application in school settings	5 Aramark restaurants	No Code (26 ago 2016 18:00)
Chilealimentos (Association of Food Industries)	Solidarity and Social Investment Fund	2016	Head of Service	To evaluate collaboration options. They want to present their programme "Healthy spaces" which has been implemented in 17 schools and covered 4.889 children, parents, teachers and other school staff as well as school kiosk owners in 5 municipalities in 2014. The programme promotes active breaks, includes nutritional education in the school curriculum, doubles physical education hours and creates healthy spaces that sell low-in-calories foods. It is effective to tackle obesity in public schools.	2 No info	No Code (30 May 2016 15:00)

Chilealimentos (Association of Food Industries)	Ministry of Health	2017	Under-secretary of Public Health	proposal to strengthen the food labelling law	3 Chilealimentos	No Code (11 Jan 2017 16:25)
Chilealimentos (Association of Food Industries)	Ministry of Health	2018	Head of the Division of Healthy Public Policies and Promotion (DIPOL)	Invitation to participate on the SDG n°2	3 Chile Crece Sano (Chilealimentos health programme)	AO001AW0491112
Chilealimentos (Association of Food Industries)	Ministry of Health	2018	Head of the Food and Nutrition Department (DIPOL)	Present Chilealimentos as a group of food companies that work directly with the ministry of Health in different areas. Members of the Review Committee of the Food Sanitary Regulations. Members of the Latin American Alliance of Food Associations (ALAIB) that participates in meetings regarding the Codex, FAO and PAHO, as well as the most important food groups in the world. They have a programme called Chile Crece Sano (Chile Grows healthy) to tackle obesity and that they have connected with Elige Vivir Sano (Chose living healthy) from the government. They ask for a meeting to show their work and propose collaborations. At the meeting they ask for impact evaluations of food labelling, discuss some products.	1 Chilealimentos	AO001AW0430536
Chilealimentos (Association of Food Industries)	Treasury	2018	Tax reform advisor	How to improve tax policies for the agricultural business sector that were defined during the previous government and that will have to be implemented by the current one.	2 Chilealimentos	AE001AW0480114
Chilealimentos (Association of Food Industries)	Ministry of Agriculture	2018	Minister	Greetings to new Minister. Discuss views and information regarding the most important topics on agroindustry for their guild (farming and commercialization of fruits and vegetables)	2 Chilealimentos	AR001AW0446133
Chilealimentos (Association of Food Industries)	Ministry of Foreign Affairs	2018	Head of General Directorate of International Economic Relations	European Union, ongoing negotiations and effects of the CAP in Chile	1 Chilealimentos, 1 Aconcagua Foods, 1 no info	AC002AW0490501
Chilealimentos (Association of Food Industries)	Ministry of Social Development	2018	Head of Elige Vivir Sano (Choose living healthy)	To present the different activities done by Chilealimentos such as 'Chile Crece Sano- Chile grows healthy'. The service explains their goals for the year, that they can sponsor industry programmes that aim to improve the quality of life of Chileans but not foods products specifically. The service also highlights the need to install water fountains in schools and Chilealimentos will analyse if they can contribute by including it to their programme. The Service is invited to participate in a national agreement led by Chilealimentos to strengthen healthy lifestyles.	3 Chilealimentos	AI008AW0439236
Chilealimentos (Association of Food Industries)	Ministry of Health	2019	Head of the Food and Nutrition Department (DIPOL)	As agreed on last OCDE meeting, they ask for a meeting to see how they can participate in designing public policies against childhood obesity as well as their participation on the Review Committee of the Food Sanitary Regulations. Also to discuss some urgent issues they have. Present a new sweetener (Allulose) to be considered in the Food Sanitary Regulations.	2 Chilealimentos	AO001AW0659581
Chilealimentos (Association of Food Industries)	Treasury	2019	Chief of Cabinet	Raise concerns and propose ideas	3 Chilealimentos	AE001AW0763417
Chilealimentos (Association of Food Industries)	Ministry of Agriculture	2019	Executive Secretary of the Chilean Agency for Food Safety and Quality (ACHIPIA)	To voice the need of the food industry of participating more actively in Codex Alimentarius. They request to be present on the next international meeting of the Codex CCLAC that will be in Chile to announce to the assembly the need of the Codex to approve and promote sensible regulations based on science and that do not create barriers to trade	2 Chilealimentos	AR001AW0742283

Chilealimentos (Association of Food Industries)	Ministry of Social Development	2019	Head of the Public-Private Cooperation Division	Know details of the public-private cooperation for an eventual joint alliance	2 Chilealimentos-Chile Crece Sano	AI007AW0576951
Chilealimentos (Association of Food Industries)	Ministry of Social Development	2019	Head of the Public-Private Cooperation Division	They say the food industry is associated with at least 9 out of 17 SDG. As representatives they request to know the mechanisms in which the private sector is being considered by the Ministry to reach the goals the country has set for 2030.	1 Chilealimentos, 1 Chile Crece Sano	AI007AW0612058
Chilealimentos (Association of Food Industries)	Ministry of Social Development	2019	Secretary of Elige Vivir Sano (Choose living healthy)	To show results that can strengthen the service's new initiative called "healthy living advisory board" from the private sector	2 Chile Crece Sano (Chilealimentos health programme)	AI008AW0753294
Chilealimentos (Association of Food Industries)	Ministry of Social Development	2019	Secretary of Elige Vivir Sano (Choose living healthy)	To see the possibility to implement Elige Vivir Sano strategies in Casablanca and to invite them to participate in FAO table to discuss SDG 2	2 Chile Crece Sano (Chilealimentos health programme)	AI008AW0641914
Chilealimentos (Association of Food Industries)	Ministry of Science	2020	Minister	To discuss challenges, goals and opportunities for collaboration	2 Chilealimentos	BD001AW0791963
Chilealimentos (Association of Food Industries)	Ministry of Social Development	2021	Secretary of Elige Vivir Sano (Choose living healthy)	Present themselves and their programme "Chile Crece Sano - Chile grows healthy". To evaluate options to make joint activities similar to previous years to strengthen the public-private alliance against obesity and associated diseases. Chile Crece Sano aims to promote healthy lifestyles and physical activity, promote consumption of an equilibrated diet and collaborate with SDG 2 (zero hunger)	3 Chilealimentos-Chile Crece Sano	AI008AW1013095
Chilealimentos (Association of Food Industries)	Ministry of Economy	2022	Minister adviser	To introduce themselves and discuss the development of the Chilean food industry	1 Chilealimentos	AH001AW1097177
Coca Cola Chile	Municipality of Coquimbo	2015	Mayor	Contributions for the local small businesses affected by the earthquake	1 Coca Cola bottling company	MU067AW0032574
Coca Cola Chile	Ministry of Health	2016	Under-secretary of Public Health	Implementation of the food labelling law	3 Coca Cola	No Code (10 Mar 2016 14:06)
Coca Cola Chile	Ministry of Economy	2017	Minister	Programme 'Almacenes de Chile' (Warehouses of Chile)	3 Coca Cola	AH001AW0343980
Coca Cola Chile	Ministry of Environment	2017	Minister	To discuss the two lines of work between Coca Cola and the government (health and environment). From the environmental point of view, they highlighted their efforts on 'returnability' and 'ecodesign', Coca Cola also mentioned the difficulties faced during the process and how they could work together to overcome them. In the health area, Coca Cola discussed their innovation efforts to provide healthier alternatives to their consumers, focusing on changing consumption trends by enhancing their low-in-sugar versions on the market and reducing the sugar content in their other beverages. They also discussed challenges and possibilities to work together to keep and strengthen these trends	4 Coca Cola	AW002AW0292088
Coca Cola Chile	Ministry of Health	2017	Under-secretary of Public Health	Reformulation of Fanta and Sprite in returnable containers. They highlight their role in collaborating with the Ministry of Health to adopt the new regulations on food labelling and in other areas. Recognises the effort in reformulating products that are a 30% of their sales volume. They have continue growing. Regulations have allowed for reformulation of products improving their composition without the need of that much non-caloric sweeteners. 65% of their portfolio have less sugar. They will do educational materials for the consumer highlighting this. They have a new mix of non-caloric sweeteners	2 Coca Cola	No Code (31 Aug 2017 15:30)

Coca Cola Chile	Ministry of Health	2018	Head of the Food and Nutrition Department (DIPOL)	To introduce the company and reformulation efforts according to law 20,606. (they don't assist)	3 Coca Cola	AO001AW0505604
Coca Cola Chile	Ministry of Health	2018	Minister	To show the progress in implementing the law 20,606	2 Coca Cola, 3 no info	AO001AW0498080
Coca Cola Chile	Municipality of Carahue	2018	Mayor	To give 4 wood kiosks to the municipality	1 Coca Cola	MU028AW0454736
Coca Cola Chile	Treasury	2018	Under-secretary of Treasury	To present the company and their programmes	4 Coca Cola	AE001AW0515825
Coca Cola Chile	Ministry of Economy	2020	Minister	Presentation of the "Mi Barrio Mi Almacén Programme" that aims to connect Neighbourhood Stores with their Clients and the Community, through the use of digital tools.	3 Coca Cola	AH001AW0849268
Coca Cola Chile	Ministry of Women and Gender equity	2020	Minister	To present their gender and diversity agenda	2 Coca Cola	BB001AW0850288
Coca Cola Chile	Ministry of Health	2021	Under-secretary of Public Health	To collaborate with the COVID-19 vaccination plan	1 Coca Cola	AO001AW0917554
Coca Cola Chile	Ministry of Social Development	2021	Under-secretary of Social Development	Social projects by Coca Cola such as 'common pots' in Bajos de Mena and Valparaiso that reactivates small business, restaurants food chains and other actors. Discuss how the industry would be affected if the idea of taxing unhealthy foods to raise revenue to manage COVID is implemented. The Secretary explains how Elige Vivir Sano works and its role in coordinating with Health and other Ministries. Also mentioned that is difficult to add more topics to what they are already doing, that they are trying to bring to this Ministry other similar projects from other Ministries and their views on the national context regarding possible taxes.	5 Coca Cola, 1 Coca Cola-Jesuit migrant service	AI008AW0958032
Coca Cola Chile	Municipality of Maipú	2021	Mayor	to introduce the company and opportunities for collaboration	3 Coca Cola, 1 Coca Cola-Jesuit migrant service	MU163AW1022521
Coca Cola Chile	Foreign investment promotion agency	2022	Head of Service	To request audience with the Minister of Economy to continue working topics discussed in a bilateral meeting at the 'Cumbre de las Americas' 2022 (Meeting of all political leaders in the American continent)	5 Coca Cola	AH001AW1148879
Coca Cola Chile	Metropolitan Regional government	2022	Regional Governor	To present themselves, to inform about their initiatives in the region and to identify potential collaboration projects.	1 Coca Cola, 1 no info	AB081AW1013257
Coca Cola Chile	Metropolitan Regional government	2022	Civil servant	To continue working on what was discussed in a previous audience with the governor regarding projects for the network of Coca-Cola storekeepers, which converge with initiatives promoted from the local government.	1 Coca Cola, 1 Coca Cola-Aconcagua cultural corporation	AB081AW1232513
Coca Cola Chile	Ministry of Women and Gender equity	2022	Chief of Staff	To present themselves to the new authorities and inform of their gender and diversity agenda and the activities being done to explore new public-private alliances	3 Coca Cola, 1 Coca Cola-Jesuit migrant service	BB001AW1096680
Ferrero	Ministry of Foreign Affairs	2016	Head of Service	Impact of new food labelling (law 20606)	1 Ferrero, 1 Italian Embassy	AC002AW0149146
Ferrero Argentina	Ministry of Economy	2022	Civil servant	Greetings	3 Ferrero Argentina	AH001AW1143488
Ferrero Argentina	Ministry of Foreign Affairs	2022	General director of bilateral economic affairs	Present the company and its presence in Chile for 31 years with 4 plants processing hazelnuts. Present their most famous products: Nutella, Ferrero	2 Ferrero Argentina	AC007AW1144946

				Rocher, Kinder and Tic Tac and inform about a 5th plant which is the results of investing 50 million dollars. Ferrero is looking for a positive relationship with the government. They highlighted how important is to have a nutrition education policy which they have already developed together with the Ministry of education of Italy. They mentioned the 'Joy of moving' programme which is already being implemented in 28 countries and they offer to share it later. The institution thanks Ferrero for the meeting and says that they share their community responsibility views and that they are seen as a good example to create productive chains.		
Ideal SA (bakery products)	Ministry of Economy	2015	Minister	To present the scope of a new project that they will be implementing in the country. Chile is a strategic location in the region so they will be building a new distribution centre in Lampa Municipality which will imply a large economic investment and new jobs	1 Ideal, 1 Ideal-Chilean National TV, 1 Uber, 1 no info	AH001AW0009736
Ideal SA (bakery products)	Municipality of Maipú	2017	Community Development office	To present their new community project on nutrition and healthy eating. The company is requested to send a budget for analysis	1 Ideal, 1 Spanish consultant company	MU163AW0248415
Ideal SA (bakery products)	Ministry of Foreign Affairs	2018	Head of General Directorate of International Economic Relations	To present the new CEO of Ideal SA, company of BIMBO Mexican Bakery Group	3 Ideal, 2 no info	AC002AW0502548
Ideal SA (bakery products)	Municipality of Chillan	2021	Mayor	To talk about their mammography programme that will be done in the region	3 Ideal	MU042AW0988923
Ideal SA (bakery products)	Municipality of Chillan Viejo	2021	Mayor	To present the plans of the company before construction of their new plant in the Municipality. Part of those plans include taking mammograms in the region plus the inauguration ceremony of their plant.	3 Ideal	MU043AW0988911
Ideal SA (bakery products)	Municipality of San Bernardo	2021	Mayor	Present the company, distribution plant and sales room. Programme Buen Vecino ('good neighbour') and how to help the local community. How the use 100% renewable energy. Possibility to sponsor a kindergarten. Help with social activities.	2 Ideal	MU281AW1009430
Ideal SA (bakery products)	Municipality of Talagante	2021	Municipal Secretary	To provide details to the mayor about their programme "Buen vecino - good neighbour" that looks to collaborate with the communities were the company is present, including Talagante. Activities for 2021 include taking mammography, community spaces development among others. The company also wants to inform the mayor about their plans to improve their plant and to live in harmony with the environment	5 Ideal	MU311AW0988978
Ideal SA (bakery products)	Municipality of Quilicura	2022	Mayor	To inform the mayor of their plans in the Municipality: to expand their plant and community initiatives for 2022.	4 Ideal	MU250AW1097153
Ideal SA (bakery products)	Municipality of Talagante	2022	Mayor	To present to the mayor the company plans for 2022 including the expansion of their Nutrabien plant (bakery products) and community initiatives	3 Ideal	MU311AW1067442
Italian Embassy	Ministry of Health	2016	Under-secretary of Public Health	Effects of the implementation of the Food labelling law and potential ideas to promote healthy lifestyles	2 Italian Embassy	No Code (22 Jun 2016 19:38)
La Feté Chocolates	Lower Chamber of Congress	2015	Congressman	Law 20,606 and its regulation guidelines	4 Chocolates del mundo	No Code (30 Nov 2015 14:00)
La Feté Chocolates	Lower Chamber of Congress	2016	Congressman	Law 20,606 and its regulation guidelines	4 Chocolates del mundo	No Code (13 Jan 2016 19:45)
La Feté Chocolates	Ministry of Economy	2016	Under-secretary of Economy	To discuss topics related to the law 20,606	4 Chocolates del mundo, 1 Chocolates del	AH001AW0198446

					mundo - Association of open TV channels of Chile	
La Feté Chocolates	Ministry of Foreign Affairs	2016	Head of Service	Talk about law 20,606 and process of internationalisation of the company	3 Chocolates del mundo, 2 consultants, 1 Association of regional TV channels	AC002AW0198463
La Feté Chocolates	Ministry of Health	2016	Head of the Food and Nutrition Department (DIPOL)	Food labelling. What can be done with La Fete Chocolates? From a legislative point of view, they cannot be excluded from the regulations because they are covered by the law 20,606. They also ask about taxes to solid foods	2 Chocolates del mundo, 1 Arcos dorados	No Code (15 Mar 2016 20:30)
La Feté Chocolates	Ministry of Health	2016	Head of the Food and Nutrition Department (DIPOL)	To personally deliver a report from INTA (National institute of food technology) on the quantity of sugar, calories, saturated fats and sodium of Arcos Dorados company products. The Ministry explains the implementation decree of the law 20,606 especially advertising restrictions. Also, how all local authorities have aligned their criteria but are still autonomous to evaluate each particular case.	1 Chocolates del mundo, 1 Arcos dorados	No Code (23 Jun 2016 13:30)
Masterfoods Chile (confectionery)	Ministry of Health	2016	Head of the Food and Nutrition Department (DIPOL)	Sanitary Food Regulations. MARS will accept the local health authority resolution regarding their products. They explain that they don't target children in their philosophy. The Ministry explains the restrictions present in the implementation decree for the food labelling law which has to be applied to all foods that have warning labels. MARS gives information explanations on how they prepare their advertising and their target audiences	1 masterfoods, 2 lawyers	No Code (19 Oct 2017 17:00)
Masterfoods Chile (confectionery)	Ministry of Health	2017	Head of the Food and Nutrition Department (DIPOL)	law 20,606. MARS have some doubts about its implementation and the use of advertising and other trademark elements. The Ministry explains the regulations and that advertising elements targeting children are different from trademark used by companies. MARS presents examples of new packages with warning labels and commits to change their advertising	2 masterfoods	No Code (18 Jan 2017 11:00)
Masterfoods Chile (confectionery)	National Consumer Service	2017	Deputy Head Legal Division	Law 20,606	2 masterfoods	AH009AW0235904
Masterfoods Chile (confectionery)	Metropolitan Regional Health Authority	2018	Regional Health Authority (SEREMI)	To discuss the company experiences and concerns as a company importing chocolates and confectionery into Chile. Discuss issues related to monitoring and importation times as they are being held for long time at customs.	4 masterfoods	AO045AW0510322
Masterfoods Chile (confectionery)	Ministry of Health	2018	Head of the Food and Nutrition Department (DIPOL)	To represent Mars in Chile and their views on public policies driven by the Ministry of health. Show that they have change artificial colours for natural colours and reduced sugars and sodium in their products. They have eliminated cartoons from their packaging	3 masterfoods	AO001AW0453169
National Advertisers Association (ANDA)	Lower Chamber of Congress	2015	Congressman	Draft bill 8026 on Food advertising	4 ANDA	No Code (5 Aug 2015 20:45)
National Advertisers Association (ANDA)	Lower Chamber of Congress	2015	Congressman	Draft bill 8026 on Food advertising	6 ANDA	No Code (22 Apr 2015 16:30)

National Advertisers Association (ANDA)	Ministry of Health	2015	Under-secretary of Public Health	Topics regarding food advertising	1 ANDA, 2 ANDA-Arcos Dorados Restaurants	No Code (6 Mar 2015 14:55)
National Advertisers Association (ANDA)	Upper Chamber of Congress	2015	Congressman	To give their views about the draft bill on Food Advertising	1 ANDA	No Code (28 Sep 2015 10:00)
National Advertisers Association (ANDA)	Upper Chamber of Congress	2015	Congressman	ANDA is received by the Congresswoman advisers. They discuss the draft bill on Food labelling	1 ANDA	No Code (1 Sep 2015 17:00)
National Advertisers Association (ANDA)	Upper Chamber of Congress	2015	Congressman	ANDA views about the Food Advertising Draft Bill	1 ANDA	No Code (16 Sep 2015 16:30)
National Advertisers Association (ANDA)	Upper Chamber of Congress	2015	Congressman	Discussion of the draft bill for Food Advertising considering that the regulations have not been evaluated and their efficiency is not clear	1 ANDA	No Code (28 Sep 2015 12:31)
National Advertisers Association (ANDA)	Ministry of Health	2016	Head of the Division of Healthy Public Policies and Promotion (DIPOL)	Advertising targeting children of foods with warning labels (law 20,606). They have doubts with "grey areas" such as colours and shimmer. Supermarkets do not receive their products with these characteristics. Colours and shimmers are not considered advertising targeting children in itself. They also show the changes to their packages to meet the law 20,606	2 no info	No Code (28 Nov 2016 18:00)
National Advertisers Association (ANDA)	Ministry of Health	2017	Minister	To present their views to the Minister that were shared in the public consultation of the implementation decree for the law 20,869 (food advertising)	1 ANDA, 1 ANDA-mining company, 1 forest company	No Code (3 Feb 2017 15:30)
National Advertisers Association (ANDA)	Ministry of Health	2018	Head of the Division of Healthy Public Policies and Promotion (DIPOL)	Food labelling law. Incorporation of healthy messages. ANDA present their concerns over the phrase "choose foods with less warning labels" as it does not meet the Spirit of the Law, they propose a new phrase that promotes the concept of healthy lifestyles.	1 ANDA	AO001AW0451077
National Advertisers Association (ANDA)	Ministry of Economy	2019	Minister	To present themselves to the new Minister and their topics of interest related to the Ministry. ANDA expresses its willingness to collaborate in common interest topics	3 ANDA	AH001AW0617495
Nestlé Chile	Ministry of Foreign Affairs	2015	Head of General Directorate of International Economic Relations	Implementation law 20,606 and food legislation	4 AB Chile, 1 AB Chile-Nestle, 1 AB Chile-CCU	AC002AW0061475
Nestlé Chile	Ministry of Work	2015	Minister	Presentation of "Youth Initiative" 2015.	3 Nestle	AL001AW0011285
Nestlé Chile	Municipality of Nuñoa	2015	Mayor	propose an activity with water slides and swimming pool for the local community	2 Nestle	MU186AW0066099
Nestlé Chile	Municipality of Osorno	2015	Mayor	To donate building from the closing plant in Osorno to build a Health Service	1 Nestle	MU191AW0060372
Nestlé Chile	National Youth Institute (INJUV)	2015	Head of Service	To present a youth employability project, INJUV will invite them to the launch of the survey of Work perceptions of young Chileans, this survey will be made available in December 2015	3 Nestle	AI005AW0010396
Nestlé Chile	Local health authority Metropolitan region	2016	Head of service	Food labelling law	2 Nestle	AO045AW0195585
Nestlé Chile	Ministry of education	2016	Under-secretary of Education	Coordinate the meeting for the Pacific Alliance Youth Initiative, which seeks to improve employability	2 Nestle	AJ001AW0208448
Nestlé Chile	Ministry of Health	2016	Head of the Division of Healthy Public Policies and Promotion (DIPOL)	Implementation of food labelling law. Nestle explains their process to adapt to the new regulations. They are worried about monitoring. The Ministry explains their monitoring mechanism and the guidelines given to the local	No info	No Code (26 May 2016 16:00)

				health authorities. Also highlights the autonomy of the local health authority. They are worried about the healthy message in advertising. The Ministry explains that will be mandatory once the decree is approved.		
Nestlé Chile	Ministry of Work	2016	Minister	Presentation of "Youth Initiative" and second meeting of the Pacific Alliance	2 Nestle	AL001AW0212027
Nestlé Chile	National Consumer Service	2016	National Director	To know the views of the National Director on how the food labelling regulations will be implemented and to explore effective communication mechanisms to inform consumers their nutrition education messages in order to continue contributing to the wellbeing and health of the country.	1 Nestle, 1 no info	AH009AW0105641
Nestlé Chile	Municipality of Macul	2017	Mayor	To present the new manager of their plant in Macul	3 Nestle	MU161AW0261722
Nestlé Chile	Municipality of Maipú	2017	Mayor	Greetings to new Mayor. Introduce the company (confectionery and cereals) that has being in the municipality for many years and always open to work together with the authorities for the benefit of the community. Introduce two programmes being implemented in the Municipality: Local Development Fund Henri Nestlé Award and Nestlé Nutrition Education Programme Healthy Children.	2 Nestle	MU163APSECM016
Nestlé Chile	Municipality of Maipú	2017	Mayor	Social projects	1 Nestle, 1 no info	MU163AW0384217
Nestlé Chile	Ministry of Agriculture	2018	Minister	Present the new Nestle plant in Teno, present the company and industry update.	3 Nestle	AR001AW0431999
Nestlé Chile	Ministry of Education	2018	Minister	Present their "Youth Initiative" in the III Pacific Alliance Youth Meeting, Healthy Children Presentation, Nestlé Chile's "Youth Initiative" aims to strengthen youth employability, linking the world of education with work. It is a global initiative present in more than 190 countries where Nestlé has operations. Thus, the company prepares the next generation of talents, contributing as a bridge between the world of education and job placement. The Ministry of Education, through Minister Gerardo Varela, has been once again invited to participate in the III Youth Meeting of the Pacific Alliance to be held in Cali, Colombia, on June 28 and 29.	3 Nestle	AJ001AW0438924
Nestlé Chile	Ministry of Health	2018	Head of the Division of Healthy Public Policies and Promotion (DIPOL)	Educational and nutritional presentation for pre-kindergarten and fourth grade children "Healthy Children" programme	3 Nestle	AO001AW0450118
Nestlé Chile	Ministry of Health	2018	Head of the Food and Nutrition Department (DIPOL)	To discuss the prohibitions regarding food labelling. Nestle mentions that they use their cartoons in packages in many countries. Potential stock break. Unclear regulation guidelines.	4 Nestle	AO001AW0487199
Nestlé Chile	Ministry of Health	2018	Department of Foods and Nutrition	Control criteria for baby formulas, the company requests precision and reduce legal uncertainty. Voice their concerns regarding adequate implementation of law 20,869 (food advertising)	5 Nestle	AO001AW0502533
Nestlé Chile	Ministry of Health	2018	Head of the Division of Healthy Public Policies and Promotion (DIPOL)	Proposal to modify the decree that regulates the mandatory healthy message in food advertising. Request of a public-private work group to define healthy messages.	1 Nestle, 2 AB Chile	AO001AW0486671
Nestlé Chile	Ministry of Social Development	2018	Head of Elige Vivir Sano (Choose living healthy)	To explain their "Healthy Children" program. Nestle requested to work together. Commitment to support the program and request sponsorship.	3 Nestle	AI008AW0472879
Nestlé Chile	Ministry of Work	2018	Minister	Greetings to new Minister. Presentation of "Youth Initiative" and III Pacific Alliance meeting	3 Nestle	AL001AW0439313

Nestlé Chile	Municipality of Macul	2018	Mayor	Presentation of BEO of Savory Chile (ice creams). Joint action plan	2 Nestle	MU161AW0423382
Nestlé Chile	Municipality of Teno	2018	Mayor	To present an environmental programme	1 Nestle, 4 no info	No Code (8 Mar 2018 15.30)
Nestlé Chile	Ministry of Agriculture	2019	National Deputy Director of the Office of Agrarian Studies and Policies	To invite and give details about a meeting between Ministers of Agriculture in Mexico as part of the Pacific Alliance	2 Nestle	AR005AW0637810
Nestlé Chile	Ministry of Economy	2019	Minister	Presentation of Nestle Chile CEO	2 Nestle	AH001AW0700613
Nestlé Chile	Ministry of Foreign Affairs	2019	Civil servant	To organise the V Youth Pacific Alliance meeting	3 Nestle	AC007AW0731931
Nestlé Chile	Ministry of Health	2019	Head of the Food and Nutrition Department (DIPOL)	Introduce ´Healthy Children programme'. Has reached 80.000 children in Chile	2 Nestle	AO001AW0700625
Nestlé Chile	Ministry of Foreign Affairs	2020	Civil servant	To make the Youth Pacific Alliance meeting if the current conditions allow it. The meeting had to be cancelled because of the pandemic and they are willing to set a new date. The Secretary says the calendar is being revised because of the pandemic	3 Nestle	AC001AW0819270
Nestlé Chile	Municipality of Teno	2020	Mayor	Nestle plant. Voluntary commitments and compliance with environmental regulations	1 Nestle, 2 no info	MU316AW0866055
Nestlé Chile	Municipality of Maipú	2021	Mayor	Introduce Nestle Plant in Maipú and discuss collaboration and projects to help the municipality	3 Nestle	MU163AW0984017
Nestlé Chile	Municipality of Osorno	2021	Mayor	Introduce Nestle Plant in Osorno and discuss collaboration and projects to help the municipality	2 Nestle	MU191AW0969767
Nestlé Chile	Los Lagos regional government	2022	Regional Governor	To present themselves to the new authority and discuss possible collaboration instances	1 Nestle	AB087AW1070952
Nestlé Chile	Metropolitan Regional government	2022	Regional Governor	To present themselves to the new authority and discuss possible collaboration instances to benefit the region	2 Nestle	AB081AW1098638
Nestlé Chile	Metropolitan Regional government	2022	Regional Governor	To present themselves to the new authority and discuss possible collaboration instances to benefit the region	2 Nestle	AB081AW1033976
Nestlé Chile	Ministry of Agriculture	2022	Minister	To present themselves and discuss projects to benefit the country and potential collaborations.	2 Nestle	AR001AW1150984
Nestlé Chile	Ministry of Economy	2022	Chief of Investments Unit	To present themselves and their "innovation, science and technology in Chile" plan	2 Nestle	AH001AW1220218
Nestlé Chile	Ministry of Health	2022	Head of the Food and Nutrition Department (DIPOL)	Declaration of healthy properties of a food and its interpretation. They want to comment deficiencies in the interpretation of healthy messages of their imported products. In their views some workers from the regional health authority (metropolitan region) are creating problems for their imports. The department informs roles and stages of the process and request to send their records to evaluate the issue	3 Nestle	AO001AW1191427
Nestlé Chile	Ministry of Science	2022	Minister	Present themselves and present their plan on innovation, science and technology in Chile	3 Nestle	BD001AW1206305
Nestlé Chile	Ministry of Work	2022	Head of Work prospecting department	To present themselves and resume public-private partnership. Nestle presented their "Youth initiatives" to improve employability. To invite the	2 Nestle	AL001AW1150967

				Minister and Secretary to a meeting of the Pacific Alliance in Mexico where Nestle and other companies participate		
Nestlé Chile	Municipality of Teno	2022	Mayor	To talk about their plant projects and collaborations to benefit the Municipality	5 Nestle	MU316AW1155814
Nestlé Chile	National School Aid and Scholarship Board	2022	Head of student nutrition department	To present themselves and discuss projects: Healthy eating programme in Peñalolen (has 3 pillars: knowledge, innovation and promoting healthy lifestyles. YouTube channel. Food consumption study (2023-2024) and Nutritional observatory (launch in 2022)	2 Nestle	AJ009AW1179722
Nestlé Chile	National Training and Employment Service	2022	Civil servant	To present themselves and discuss projects to benefit the country and potential collaborations.	2 Nestle	AL007AW1171780
Nestlé Chile	National Youth Institute (INJUV)	2022	Institutional relations unit manager	To present themselves and talk about projects for the benefit of the country and possible collaborations	2 Nestle	AI005AW1171775
Red Bull	Ministry of Health	2017	Head of the Food and Nutrition Department (DIPOL)	To present their views regarding changes to the regulations on supplement and foods for athletes. The ministry explains the formal process to modify the Food Sanitary Regulations. They are worried that energy drinks, which are athlete food would be forbidden.	4 Red Bull	No Code (10 Aug 2017 12:30)
Red Bull	Ministry of Health	2017	Head of the Food and Nutrition Department (DIPOL)	Potential changes to the Food Sanitary Regulations regarding food supplements. Chile is the 2nd most important country in Latin America for Red Bull sales. The Ministry explains that the changes are to better organise the regulations and their rationale. They also ask about draft bill to be send to Congress	1 Red Bull	No Code (30 Jan 2017 8:00)
Red Bull	National Consumer Service	2017	Head of Service	Red Bull Latam wants to meet with the Head of service to discuss the "labelling and nutritional information for energy drinks" study released by the service that week to show them all their advances on several topics covered by that study	2 Red Bull, 1 Red Bull-Ecusa drinks, 1 Ecusa drinks	AH009AW0378780
Red Bull	Ministry of Culture	2018	Civil servant	Request to meet the Secretary to support cultural activities being done by the Ministry. Red Bull representatives do not arrive to the meeting	3 Red Bull	BC001AW0558314
Red Bull	Ministry of Culture	2018	Under-secretary of Culture and Arts	To explore joint activities in the cultural sector	3 Red Bull	BC001AW0581922
Red Bull	Ministry of Economy	2018	Under-secretary of Tourism	To inform about their activities to promote tourism	3 Red Bull	AH011AW0557451
Red Bull	Ministry of Health	2018	Head of the Food and Nutrition Department (DIPOL)	To ask about regulations for food supplements and the implementation second stage of the food labelling law regarding monitoring intensity. The ministry responds that all products are monitored in the same way according to the Sanitary Food Regulations code	3 Red Bull-Ecusa Drinks	AO001AW0462119
Red Bull	Ministry of Health	2019	Head of the Food and Nutrition Department (DIPOL)	Collaborate and provide background information on queries, regulations, standards and issues related to energy drinks. Company gives a proposal, the Ministry tells them they should follow the appropriate mechanisms. Ministry clarifies how energy drinks are classified (as drinks)	4 Red Bull	AO001AW0730357
Red Bull	National Consumer Service	2019	Audit Deputy Director	Collaborate and provide background information on queries, regulations, standards and issues related to energy drinks	3 Red Bull	AH009AW0730390
Santiago Chamber of Commerce	Ministry of Health	2017	Head of the Food and Nutrition Department (DIPOL)	Suggestions to improve the food labelling law and to make more efficient the management of import companies (labelling of foods being expensive and a barrier for importation of products. Also places the international producer in disadvantage compared to the national producer). They also ask about the evaluation of the first 6 months on the law. Problems with logos	1 Santiago Chamber of Commerce, 2 no info	No Code (31 Jan 2017 8:00)

				that are attractive to children. Modify the regulations by categories and portion sizes. It is suggested that they present their proposal to the Secretary of public health		
Santiago Chamber of Commerce	Ministry of Health	2018	Head of the Food and Nutrition Department (DIPOL)	Concern about the long periods in processing imported products. Difficulty to follow the importation process. Concern in adapting imported products to meet food labelling standards (law 20,606). Ministry of Health has shown willingness to address proposals to modernize import regulations	2 Santiago Chamber of Commerce	AO001AW0553090
SOFOFA (Federation of Chilean Industry)	Ministry of Health	2015	Minister	Food labelling. Psychosocial and work risks. Disinfectants	1 SOFOFA	No Code (19 Feb 2015 18:00)
SOFOFA (Federation of Chilean Industry)	Presidency	2016	Minister Secretary General of the Presidency	Food labelling	1 SOFOFA, 1 AB Chile-SOFOFA	AF001AW0069792
Soprole (yogurts, milks)	Municipality of San Bernardo	2021	Mayor	To present the company. Inform that 16% of workers belong to the municipality. Mayor thanks the company for their support. Company offers help with the programme Buen Vecino (good neighbour), more jobs for the neighbours, support to the community, mother day and children's day celebration. In environmental matters, they talk about packages of their products and work with Techo Chile (building programme) for house siding with eco-wood and plastic. Offer to install a playground using recycled plastic. Idea of creating an agenda to work together. Mayor instructs public servant in the municipality to keep working with the company.	3 Soprole	MU281AW0989361
Tresmontes Lucchetti	National Indigenous Development Corporation	2021	Head of service	Potential of Tresmontes Lucchetti SA for collaborations and implementation of public-private projects	2 Tresmontes Lucchetti	AI002AW0922642
Unilever	Ministry of Health	2016	Head of the Food and Nutrition Department (DIPOL)	Implementation of the food labelling law and nutritional compatibilities. The Ministry explains the formal mechanism to request modifications to the regulation	2 Unilever	No Code (30 Aug 2016 19:00)
Unilever	Ministry of Health	2017	Head of the Food and Nutrition Department (DIPOL)	Doubts about the implementation of law 20,606 regarding claims and cholesterol in margarine. The Ministry explains regulations	3 Unilever	No Code (13 Jan 2017 8:00)
Watts (juices)	Ministry of Education	2016	Head of School Feeding Programme - National school aid and scholarship board	To request information on new law [food labelling], to present new products that could fit the requirements for PAE (School feeding programme)	1 Watts, 1 no info	AJ009AW0110542
ILSI (international Life Sciences Institute) Sur-Andino	Ministry of Health	2015	Undersecretary of Public Health	ILSI worldwide, has as its mandate to serve and support the dialogue between governments, academia and the industry to benefit the health of the people. Prioritising the Ministries of Health, Agriculture and Environment. Because of this, ILSI requests a meeting to present their work and learn the current health needs in which they can support the Ministry.	2 ILSI	No Code (9 April 2015 6:30)
Chilean Supermarket Union Association	Ministry of Health	2016	Civil servant	Implementation of food labelling law. MINSAL informs they do not authorise specific products. MINSAL recognised efforts in advertising. Association has doubts regarding use of brands and its monitoring. MINSAL informs that any changes will be informed through the Sanitary Food Regulations, that document is the guide used by inspectors. Association also asks to clarify doubts regarding advertising regulations.	8 supermarket association	No code (26 Dic 2016 12:10)
DANONE	Ministry of Health	2020	Civil servant	Request to participate in Codex Alimentarius and committees that discuss food regulations particularly regarding new ingredients and products. Cross	1 Danone	No code (29 Oct 2020 10:30)

				advertising and alignment of the WHO breastfeeding code by the industry in Chile. Discussion of baby formulas.		
DANONE	Ministry of Health	2020	Civil servant	To understand and use the regulations regarding infant formulas. They have concerns regarding advertising of infant formulas in tv, medical samples and selling of products in informal markets.	1 Danone	No code (6 Aug 2020 11:00)
DANONE	Ministry of Health	2018	Civil servant	Danone will continue in the country but will not do anymore advertising of their products. This is a budgetary decision, not a commercial one. They remain committed to the country but have not certainty about future decisions. They may retire their products from the country. They request a letter of concern from the Minister of Health. MINSAL explain that these topics are more related to the Ministry of Economy and that they should talk with them. Also, that they must fulfil their contractual commitments	2 Danone	No code (17 Jan 2018 10:00)
PepsiCo	Ministry of Environment	2018	Civil servant	Sustainability strategy PepsiCo 2018-2019 and new compostable packaging	5 Evercrisp Snack products SA	No code (9 Oct 2018 15:00)
PepsiCo	National School Aid and Scholarship Board	2019	Civil servant	To present Contrapeso plan PepsiCo is implementing in schools in Cerrillos and Renca municipalities. It is about healthy eating. Work through the food network with the communities with different programmes managed by the foundation.	1 'private interest' manager	No code (12 Feb 2019 11:30)
PepsiCo	Pichilemu Municipality	2015	Mayor	Since 2013, Casa de la Paz (house of peace) Foundation has worked with the municipality with PepsiCo, AprenDAR and ActivaRSE programmes. From that work, they have generated strong ties with school communities.	1 Casa de la Paz Foundation	No code (10 Dic 2015 15:15)
PepsiCo	Renca Municipality	2017	Civil servant	to present FUNDES foundation and a programme that together with PepsiCo aims to support a group of small grocery stores to make their businesses sustainable and profitable over time	1 'private interest' manager	no code (18 Jul 2017 12:00)
