# Supplementary material for: Analysis of public records of lobbying practices of the ultra-processed sugary food and drink industries in Chile: a qualitative study
Source: Lancet Reg Health Am. 2024 Jun 9;35:100794. doi: 10.1016/j.lana.2024.100794 (PMC11294830; doi:10.1016/j.lana.2024.100794)
Supplement: Abstract in Spanish [file mmc2.docx]

**Resumen**

Introducción

Dado el rol de los determinantes comerciales en el consumo de azúcar y la salud, este estudio tuvo como objetivo describir las prácticas de lobby de las industrias de alimentos y bebidas ultra-procesadas altas en azúcar en Chile entre 2014 y 2022.

Métodos

Se identificaron a través del Registro de Lobby de Chile reuniones entre industrias de alimentos y bebidas ultra-procesadas altas en azúcar y actores relacionados y funcionarios del gobierno. Actores relevantes se identificaron inicialmente en función de su participación en el mercado y se ampliaron iterativamente en función de la información obtenida de las reuniones. El análisis cualitativo siguió un enfoque deductivo-inductivo utilizando el “Modelo de Actividad Política Corporativa” para identificar y clasificar objetivos, estrategias de acción y de discurso.

Resultados

De los 237 registros identificados, los Ministerios de Salud, Desarrollo Social y Economía fueron los más solicitados para reuniones de lobby. Representantes de la industria trataron de lograr sus objetivos a corto y largo plazo dirigiéndose a una amplia gama de autoridades, incluidos ministros y subsecretarios, utilizando diversas estrategias. Las estrategias discursivas se centraron en presentar a las industrias de alimentos y bebidas altas en azúcar como actores políticos socialmente responsables y legítimos, y criticaron las iniciativas de salud pública como “soluciones deficientes”. Estrategias de acción se enfocaron en acceder e influir en la formulación de políticas y fomentar la reputación corporativa.

Interpretación

Las industrias de alimentos y bebidas altas en azúcar realizaron lobby frecuentemente entre 2014 y 2022, un período en el que se discutían políticas de salud pública de gran envergadura en Chile. Las estrategias de lobby variaron para cumplir los objetivos de la industria y se dirigieron a una amplia gama de instituciones gubernamentales, incluidos ministros y funcionarios de alto rango. Se necesitan con urgencia regulaciones más estrictas para detener la influencia inapropiada de la industria en la formulación de políticas de salud pública.

Financiamiento

Agencia Nacional de Investigación y Desarrollo (Chile)-Beca de Doctorado en el extranjero. University College London – costos de Open Access
